# Bone aging: a paradigm of multiscale degeneration and targeted rejuvenation

**DOI:** 10.1038/s41413-026-00582-w

**Published:** 2026-09-14

**Authors:** Kangpeng Li, Chenyuan Gao, Ti Zhang, Cheng Wang, Xiao Geng, Xinguang Wang, Hua Tian

**Affiliations:** Department of Orthopedics, Peking University Third Hospital; Engineering Research Center of Bone and Joint Precision Medicine, Ministry of Education, Beijing, 100191 China

**Keywords:** Metabolic disorders, Bone

## Abstract

Bone aging is not merely a process of bone mass loss but rather a multi-level, networked degenerative phenomenon spanning molecular, cellular, tissue, and system levels. With the accelerating pace of global population aging, bone degenerative diseases have emerged as a major public health challenge. Consequently, there is an urgent need to elucidate the underlying mechanisms from an integrated perspective and to develop innovative intervention strategies. This review systematically synthesizes the comprehensive landscape of bone aging. At the macroscopic level, bone aging is characterized by declines in bone mass, microstructure, and biomechanical properties, ultimately leading to increased fragility. At the microscopic level, its pathogenesis involves dysfunction across multiple layers. Molecular mechanisms include genetic and epigenetic alterations, oxidative stress, the senescence-associated secretory phenotype, and dysregulation of key signaling pathways. Cellular level changes involve telomere attrition, mitochondrial and endoplasmic reticulum dysfunction, impaired proteostasis, altered nutrient sensing, and cellular senescence. At the microenvironmental and systemic levels, contributing factors include chronic inflammation, hormonal changes, and extracellular matrix remodeling. Correspondingly, intervention strategies include non-pharmacological approaches, such as dietary optimization, physical activity, and avoidance of alcohol and tobacco, as well as pharmacological therapies, including antiresorptive agents, anabolic bone-forming drugs, and senolytics. Emerging therapeutic avenues include regenerative medicine, gene therapy, and tissue engineering. Future research should focus on constructing a multi-omics-integrated bone aging atlas, developing bone organoids and computational models, establishing multimodal risk assessment systems, advancing precision therapies that target aging-related pathways, and promoting prevention-centered public health strategies. This review aims to provide a systematic framework for understanding the complex pathophysiology of bone aging and to provide key insights and directions for future interdisciplinary collaborations, translational research, and clinical interventions.

## Introduction

**Fig. 1:**
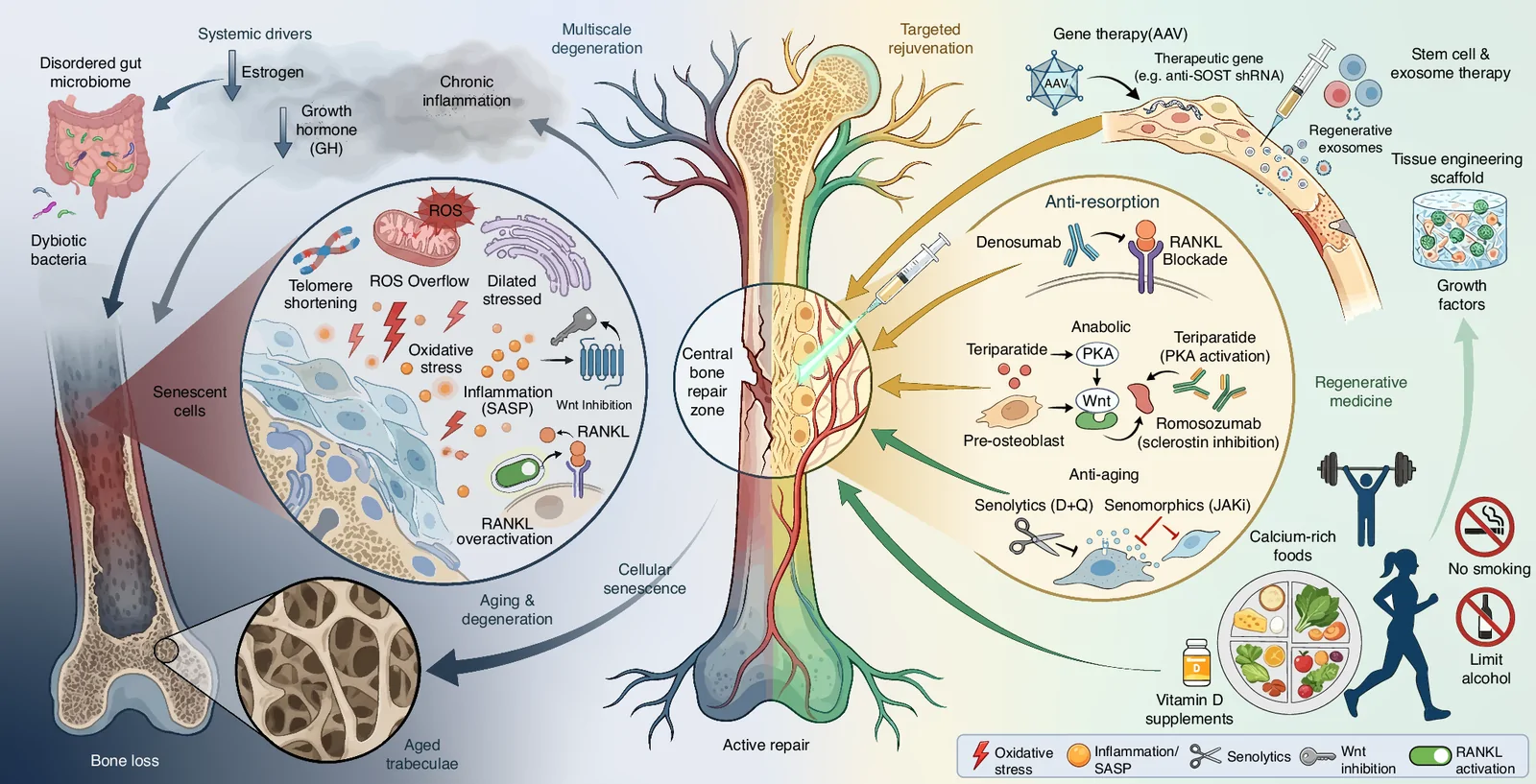
Bone aging: a multiscale degenerative cascade and targeted rejuvenation. Taking a femur in the process of repair as a life metaphor, this figure summarizes the multi-level degenerative cascade of bone aging and the targeted regeneration strategy. The composition uses a contrast narrative between the left and right: the left side depicts the multi-scale destructive forces from macroscopic bone loss, microscopic cellular aging to systems-driven. On the right side, the multi-level recovery strategy from basic nutrition and exercise (lifestyle factors appear here in a conceptual/foundational role, not as mechanistic drivers or detailed intervention targets, see Figs. 5 and 6 for those distinct perspectives), precision medicine intervention to cutting-edge regenerative therapies are systematically displayed. The focal repair zone of the central skeleton symbolizes the central role of targeted intervention, highlighting the dynamic balance of damage and repair and the transformation potential. The illustration is conceptual and metaphorical, not a literal anatomical rendering; vascular elements are stylized to represent hierarchical therapeutic strategies rather than physiological vessel caliber

The skeletal system is not merely an inert structural scaffold but a living organ characterized by a highly active metabolism and continuous dynamic remodeling. Central to this process is bone remodeling, a tightly regulated and precisely coupled sequence of bone resorption mediated by osteoclasts and bone formation driven by osteoblasts. This process is essential not only for maintaining mineral homeostasis but also for repairing microdamage, adapting to changes in the mechanical environment, and preserving skeletal integrity and biomechanical function. Disruption of the balance between bone resorption and bone formation directly compromises bone quality and strength (Fig. 1).

With advancing age, the regulatory capacity of this finely tuned system inevitably declines, resulting in progressive and systemic skeletal deterioration, a process referred to as bone aging. Bone aging is characterized not only by a reduction in bone mass but also by spatiotemporal dysregulation of bone remodeling and destructive changes in bone microstructure. The most immediate clinical consequence is a markedly increased risk of osteoporosis. According to the International Osteoporosis Foundation, an osteoporotic fracture occurs every three seconds worldwide.^1^ In the context of accelerated global population aging, the rising incidence of disability and mortality associated with fragility fractures, particularly of the hip and vertebrae, along with the substantial medical and social care burden they impose, represents a major public health challenge. Beyond its clinical implications, the study of bone aging also offers a valuable model for understanding coordinated aging processes across multiple organ systems.

Against this background, this review seeks to move beyond the traditional dualistic framework of bone resorption-formation imbalance and to systematically delineate bone aging as a multi-level, networked degenerative process. We first summarize the phenotypic manifestations of bone aging, ranging from declines in macroscopic mechanical properties to deterioration of microstructural integrity. We then examine the core mechanisms underlying the spatiotemporal dysregulation of bone remodeling units at the molecular and cellular levels. Finally, we integrate recent advances in therapeutic and preventive strategies targeting bone aging. By synthesizing current concepts and emerging evidence, this review aims to provide an integrated framework for understanding the pathophysiology of bone aging and to offer a solid theoretical foundation and clear research directions for the development of innovative strategies to prevent and treat age-related skeletal disorders and restore bone homeostasis.

## Macroscopic characteristics: progressive deterioration of bone mass, structure, and biomechanics

### Bone mass

**Fig. 2:**
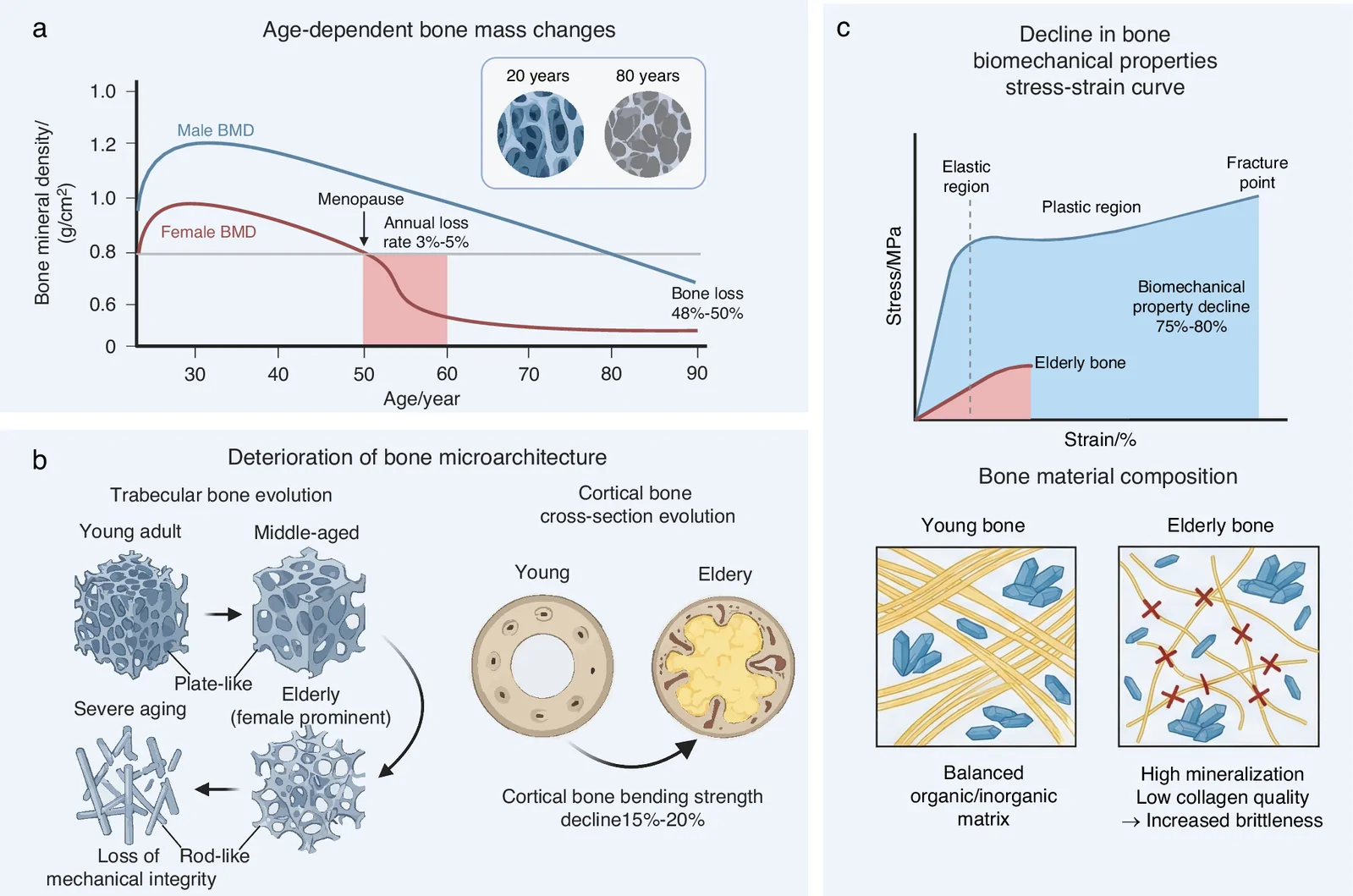
Macroscopic deterioration in bone aging. **a** Age-related trends in bone mineral density in men and women, particularly the rapid bone loss in women after menopause, are supplemented by a simulated comparison of trabecular bone mass. **b** The degeneration process of cancellous bone from a young thick plate-like network to an old sparse broken rod-like structure through a sequence diagram, and the change of cortical bone with a thinner cross-section and increased porosity. **c** The upper stress-strain curve illustrates decreased elastic modulus and increased brittleness in aged bone compared with young bone. The inferior diagram depicts age-related shifts in bone material composition: young bone maintains a balanced organic/inorganic matrix, whereas elderly bone exhibits increased mineralization and reduced collagen quality, leading to increased brittleness and fracture susceptibility

Bone mass refers to the total mineral content within bone tissue. Dynamic changes in bone mass throughout life are governed by bone remodeling, a tightly coordinated process involving osteoblast-mediated bone formation and osteoclast-mediated bone resorption. This process underlies the characteristic trajectory of bone mass across the lifespan—growth, peak, maintenance, and loss (Fig. 2).

Bone mass increases rapidly from birth, with peak bone mass achieved between 25 and 30 years of age, showing significant sex differences: males reach peak later (around 30 years) and at a higher level (lumbar spine BMD ~ 1.2 g/cm²) than females, who peak 2–3 years earlier at ~1.0 g/cm². After age 40, both sexes lose bone, but women experience an accelerated phase during menopause, with annual bone loss reaching 3%–5%—approximately twice that of age-matched men.^2^ Cumulative trabecular bone loss from 20 to 80 years is ~40% in women versus ~25% in men.^3^ Earlier studies indicate that in women, vertebral bone loss begins in early adulthood and progresses linearly, whereas appendicular atrophy typically starts around age 50, accelerates between 51 and 65 years, and slows thereafter. Lifetime vertebral loss reaches ~47%, compared with ~30% in the mid-radius and ~39% in the distal radius. In men, age-related bone loss at both axial and appendicular sites is comparatively modest or statistically insignificant.^4^ Substantial interindividual variability in bone aging rates suggests a strong genetic component.^5^

Despite the widespread acceptance of these general trends, several areas remain debated. For instance, the relative contributions of estrogen decline versus other age-related factors to the menopausal acceleration of bone loss are not fully resolved, and cross-sectional estimates of cumulative loss may obscure substantial individual variability in longitudinal trajectories. Furthermore, whether age-related bone loss in men is truly modest or simply underdetected due to less frequent study of male cohorts at advanced ages remains contested. From a therapeutic standpoint, these uncertainties complicate decisions about when to initiate pharmacologic intervention in perimenopausal women versus older men, as most clinical trial evidence derives from postmenopausal women, with limited guidance for sex-specific or frailty-stratified approaches. Identifying these knowledge gaps is essential for prioritizing future research into personalized fracture prevention.

### Bone microstructure

Bone microstructure, a central determinant of bone quality, refers to the microscopic organization and structural characteristics of bone tissue.^6^ At the time peak bone mass is achieved, bone microarchitecture is relatively stable. With advancing age, however, progressive deterioration occurs, particularly within the trabecular bone. Age-related increases in relative or absolute bone resorption lead to a reduction in trabecular number, thickness, and connectivity. Trabeculae become thinned, perforated, and ultimately fragmented, transforming the original interconnected plate-like network structure into isolated rod-like structures. This architectural disruption markedly compromises mechanical integrity and load-bearing capacity, particularly in cancellous bone-rich regions such as the vertebral bodies and hip.^7^

Age-related changes in cortical bone are equally consequential. Cortical thickness gradually decreases while porosity increases, driven by enhanced endocortical resorption and insufficient periosteal bone formation, along with enlargement of Haversian and Volkmann canals and increased intramedullary fat accumulation.^8–10^ This cortical porosification reduces bone density and substantially weakens resistance to bending and torsional forces.^6^ Concurrently, aging dysregulates bone remodeling and microdamage repair: resorption cavities are inadequately refilled, leading to net bone loss per remodeling cycle, while declining repair capacity permits microdamage accumulation. Within a compromised microarchitectural framework, these microdamages coalesce into microcracks, ultimately precipitating fragility fractures.^11^

While the above microarchitectural deterioration model is widely accepted, several important uncertainties remain. First, causal relationships between specific microstructural parameters and fracture risk are often inferred from cross-sectional histomorphometry or high-resolution imaging, but longitudinal evidence tracking the same individuals over time remains sparse. Second, although dysregulated remodeling and microdamage accumulation are implicated in age-related fragility, the threshold at which microdamage becomes clinically relevant—and whether it can be modified independently of bone mineral density—remains poorly defined. Critically, the majority of microstructural data derive from postmenopausal women, limiting extrapolation to men, younger populations, or those with chronic diseases, where bone microarchitecture may follow distinct, non-classical aging trajectories.

### Bone biomechanics

The biomechanical deterioration of aged bone extends beyond simple declines in BMD, involving complex alterations in material properties and fracture behavior. While it is well established that aging reduces bone strength, stiffness, and energy absorption capacity, recent advances have shifted focus toward understanding the multiscale origins of brittleness. Studies examining age-related changes in vertebral trabecular bone biomechanics have shown that between 20 and 80 years of age, BMD decreased by approximately 48%-50%, whereas vertical stress resistance, stiffness, and energy absorption capacity decreased by 75%-80%. Biomechanical performance in both vertical and horizontal orientations exhibits a strong positive correlation with BMD.^12^ In cortical bone, bending strength decreases by approximately 15%-20% between 35 and 70 years of age, while compressive strength decreases by nearly 50%. In parallel, fracture energy absorption is markedly reduced reflecting increased bone brittleness.^13^ These changes are associated with alterations in bone composition. Under normal conditions, the organic matrix and inorganic mineral components are well-balanced; however, aging shifts the balance toward higher mineral content and reduced collagen quality, thereby increasing bone stiffness at the expense of toughness.^14^

Two emerging concepts are worthy of attention. First, the spatiotemporal heterogeneity of tissue mineralization—rather than mean mineral content—has been identified as a critical determinant of fracture resistance. Studies reveal that aged bone exhibits increased mineral heterogeneity at the lamellar level, creating local stress concentrators that facilitate microcrack propagation.^15–17^ Second, the quality and cross-linking profile of type I collagen are now recognized as equally important as mineral content. Age-related accumulation of advanced glycation end products (AGEs) induces non-enzymatic cross-links that restrict collagen fibril sliding, a key energy-dissipation mechanism.^18,19^ A critical unresolved question is whether current anti-osteoporotic therapies—which primarily target bone mass—meaningfully restore the organic matrix quality and toughness of aged bone. Addressing this gap will require integrating biomechanical testing with longitudinal clinical cohorts and advanced imaging biomarkers.

## Microscopic features: from molecules and cells to the microenvironment

### Molecular level

#### Genetics

**Fig. 3:**
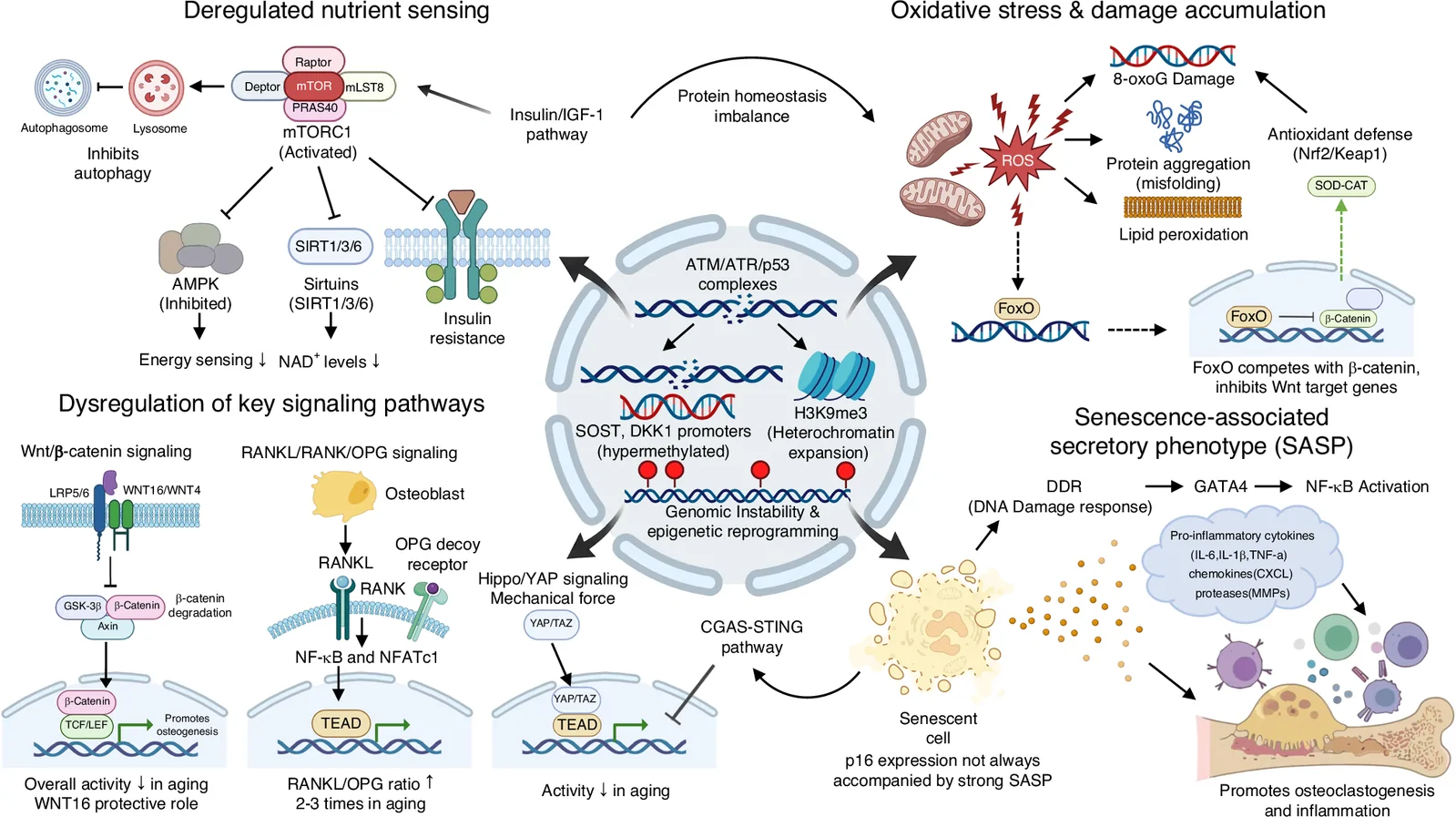
Molecular network of bone aging. The radial network Figure illustrates the core molecular mechanism network driving bone aging. The central nucleus highlights genomic instability with epigenetic reprogramming. Four interconnected pathways were identified: 1) oxidative stress leads to biomacromolecule damage and inhibits osteogenic Wnt/β-catenin signaling; 2) aging-associated secretory phenotypes triggered by the DNA damage response disrupt the bone microenvironment by releasing proinflammatory cytokines; 3) imbalance of key signaling pathways, including decreased Wnt activity, increased RANKL/OPG ratio, and inhibited Hippo/YAP pathway; 4) dysregulated nutrition sensing

The genetic mechanisms underlying bone aging are often portrayed as a complex, multi-dimensional network integrating aging biology with skeletal regulatory processes (Fig. 3). However, direct genetic studies focused explicitly on bone aging remain limited, and most current knowledge is extrapolated from transcriptomic analyses of mixed aging models.^20–22^ While features such as DNA damage accumulation, metabolic reprogramming, and senescence-associated secretory phenotype (SASP) expression are consistently described,^23,24^ whether these events drive bone aging or merely accompany it remains unclear. Functional evidence is largely confined to mouse models carrying deficiencies in DNA repair genes (e.g., XPD, ATM, ERCC1-XPF), which produce premature aging and osteoporosis-like phenotypes,^25–28^ but the translational relevance of these severe genetic disruptions to human physiological bone aging is questionable. Conversely, enhanced DNA repair via Ku70 or DNA-PKCs has been shown to protect bone mass in animal models, yet human validation is lacking.^29^

Genome-wide association studies have repeatedly implicated the Wnt signaling pathway (LRP4/5, SOST, WNT16/4) in BMD and fracture risk,^30–32^ but these associations explain only a small fraction of heritable variation, and the causal variants at most loci remain unidentified. Similarly, transcriptomic profiling has nominated genes such as ACSL3, DKK1, and SOST, while the SenMayo gene set has confirmed upregulation of CDKN1A/p21Cip1 and SASP factors in aged human bone at single-cell resolution.^33^ These descriptive inventories, however, do not establish causality, nor do they clarify whether the same senescence markers are consistently expressed across different skeletal sites, cell types, or disease states (e.g., diabetes, chronic kidney disease). Furthermore, proposed links between bone aging and lipid metabolism remain correlational,^34^ with no interventional evidence that modifying lipid pathways alters skeletal aging. A critical unmet need is therefore to move from transcriptomic cataloging toward functional validation—ideally in human tissue—and to determine which of these genetic and molecular features are amenable to therapeutic targeting.

#### Epigenetics

Epigenetic regulation—including DNA methylation, histone modifications, non-coding RNAs, and chromatin remodeling—is widely invoked as a central mechanism in bone aging.^35^

DNA methylation studies have reported associations between accelerated epigenetic aging and fracture risk in twin cohorts,^36^ and epigenome-wide association studies have identified age-related differentially methylated loci such as NOP56, TNXB, PDE8A, and AKAP12.^37^ Yet these associations are largely derived from bulk tissue analyses, which cannot distinguish whether methylation changes drive bone aging or merely reflect it. Tissue-specific comparisons between bone and blood have revealed different methylation patterns at key metabolic regulators (MEPE, SOST, WIF1, DKK1),^38^ highlighting a critical but unresolved question: to what extent can blood-based epigenetic markers serve as valid proxies for skeletal aging? The proposed development of tissue-specific epigenetic clocks^39^ is a reasonable response to this problem, but such clocks remain technically challenging and not yet validated in independent cohorts.

Non-coding RNA studies, predominantly in ovariectomized mouse models, have implicated miR-29a-3p, miR-29c-3p, and miR-874-3p in suppressing osteogenic differentiation.^40,41^ Whether these findings translate to human physiological bone aging—or to male skeletal aging—is unclear, as most rodent studies use acute hormone withdrawal rather than gradual age-related decline.

Histone modifiers such as KDM3A, KDM4C, and BRD9 have been linked to mesenchymal stem cell senescence and osteogenesis,^42,43^ but the majority of evidence comes from in vitro or overexpression models, with little understanding of how these modifications integrate with other epigenetic layers or vary across skeletal sites. Finally, SIRT3 S-sulfhydration has been shown to counteract BMSC senescence in animal models,^44^ but whether this represents a tractable therapeutic target in aged humans remains speculative. Collectively, the epigenetic literature on bone aging provides a rich descriptive foundation, but causal validation, tissue-specific benchmarking, and translational evidence lag considerably behind.

#### Oxidative stress

Bone aging is closely associated with oxidative stress, with reactive oxygen species (ROS) serving as a core driver. Excessive ROS directly damage cellular components and activate DNA damage response pathways, leading to cell-cycle arrest and senescence. Oxidative stress-induced senescence of BMSCs has been identified as a significant factor in postmenopausal osteoporosis.^45^ BMSCs isolated from OVX mice exhibit elevated ROS levels and premature senescence, both of which can be rescued by the phytoestrogen genistein.^45^

ROS exerts dual effects within the skeletal system. At physiological levels, osteoclast-derived ROS are essential for bone remodeling and promote osteoclast differentiation via activation of RANKL signaling.^46^ However, excessive ROS disrupts skeletal homeostasis by inhibiting osteogenic and mineralization in osteoblasts^47^ while simultaneously enhancing osteoclastogenesis through mechanisms such as increasing the RANKL/OPG ratio,^48^ ultimately leading to net bone loss. Similar ROS-driven mechanisms have been invoked in osteoarthritis progression,^49^ but whether therapeutic ROS reduction would benefit both bone and cartilage or exert site-specific effects is unknown.

Therapeutic strategies targeting oxidative stress have demonstrated promising effects. Pyrroloquinoline quinone alleviates age-related osteoporosis by activating the Nrf2 antioxidant pathway, reducing Rankl expression in osteoblasts, and inhibiting osteocyte senescence.^50^ Ganoderic acid D delays BMSC senescence by enhancing antioxidant enzyme activity and reducing advanced glycation end product accumulation.^51^ Modulation of deacetylases such as SIRT1 and SIRT6 has also shown protective effects against chondrocyte senescence.^52,53^ However, virtually all of these findings are at the pre-clinical stage, with no large-scale human trials demonstrating fracture reduction or long-term safety.

#### Senescence-associated secretory phenotype

The SASP comprises a complex, diverse array of bioactive factors secreted by senescent cells and functions as a potent signaling system that reshapes the tissue microenvironment. SASP components include pro-inflammatory cytokines, chemokines, growth factors, and proteases. Beyond the general features, two additional dimensions of SASP biology warrant particular attention in the context of bone aging: its tissue-specific signature and age-dependent dynamics.

In the skeletal system, the SASP profile differs markedly from that of other aging tissues. For instance, while senescent fibroblasts in skin and lung predominantly secrete IL-6 and IL-8,^54,55^ senescent osteocytes and bone marrow macrophages show heightened expression of osteoclastogenic factors such as RANKL, M-CSF, and IL-11,^56^ alongside canonical inflammatory cytokines. This pro-resorptive bias of the bone SASP reflects the unique evolutionary pressure on the skeleton as a load-bearing, metabolically active organ. SASP production is also cell-type specific. Senescent osteocytes and bone marrow macrophage-lineage cells represent major sources of SASP, whereas osteoblasts and their progenitors contribute relatively less.^57,58^ This heterogeneity complicates SASP identification at the single-cell level, highlighting the utility of standardized tools such as SenMayo aids for precise characterization.^33^

Equally important is the biphasic nature of SASP across the lifespan.^56^ In youth, low-level, transient SASP serves homeostatic functions—recruiting immune cells for tissue repair, coordinating remodeling after microdamage, and maintaining hematopoietic stem cell niche dynamics. With advancing age, however, the accumulation of senescent cells and persistent DNA damage transforms SASP into a chronic, self-amplifying inflammatory network.^59^ This age-related SASP escalation in bone directly correlates with cortical porosity, trabecular fragmentation, and fracture risk, independent of bone mineral density.

Understanding these tissue- and age-specific SASP features is not merely academic; it carries direct therapeutic implications. Suppression of SASP signaling using Janus kinase inhibitors (JAKi) effectively attenuates age-related bone loss.^60^ Similarly, the tyrosine kinase inhibitor cabozantinib prevents estrogen deficiency-induced bone loss in mice by inhibiting SASP secretion from senescent osteoblasts and their progenitors.^61^ A senolytic or senomorphic strategy effective in clearing senescent cells or suppressing SASP in bone may not translate to the brain or kidney, and vice versa. Therefore, future studies must systematically map the SASP atlas across organs and ages to enable precision targeting.

#### Key signaling pathways

Bone aging is governed by a complex and interconnected network of signaling pathways that collectively regulate skeletal homeostasis and degeneration.

The Wnt/β-catenin pathway is widely recognized as a central regulator of skeletal development and maintenance, essential for osteoblast lineage commitment. While overall Wnt signaling appears downregulated in the bones of elderly women,^62^ certain pathway members exert protective effects: osteoblast-derived WNT16 inhibits osteoclast RANK signaling, and WNT4 promotes bone formation while suppressing resorption and inflammation.^63,64^ These findings have prompted therapeutic targeting of Wnt inhibitors, most notably anti-sclerostin antibodies, which have shown positive clinical results.^65^ However, key uncertainties remain regarding the relative contribution of canonical versus non-canonical signaling in human bone aging, the cardiovascular safety of pathway activation, and whether interactions with oxidative stress or age-related decline of Gαi1/3 are causally significant in human skeletal aging.^66,67^

The RANKL/RANK/OPG axis serves as the principal regulator of osteoclast differentiation and bone resorption. Aging is associated with a two- to threefold increase in the RANKL/OPG ratio, significantly enhancing osteoclast activation, particularly under estrogen-deficient conditions. RANKL binding to RANK activates NF-κB and MAPK signaling via TRAF6, inducing NFATc1 expression and driving osteoclastogenesis.^68^ Therefore, this axis represents a key therapeutic target for intervening in bone resorption.

The Hippo signaling pathway, a key sensor of mechanical forces and cellular stress, regulates aging in a cell type-dependent manner. Activity of its downstream effectors, YAP and TAZ typically declines with age. YAP is essential for maintaining osteogenic potential in mesenchymal stem cells and suppressing adipogenic differentiation.^69^ Moreover, YAP/TAZ exert senomorphic effects by suppressing the cGAS-STING pathway and display extensive crosstalk with Wnt and TGF-β signaling.^70,71^

Fibroblast Growth Factor (FGF) signaling exhibits diverse and context-dependent effects on skeletal aging. FGF2 promotes bone growth and repair via activation of Wnt/β-catenin and BMP2 pathways.^72^ FGF19 exerts a protective effect on bone density by regulating bile acid and lipid metabolism.^73,74^ Conversely, excessive FGF23 disrupts phosphate homeostasis and reduces bone density.^75,76^ The relationship between FGF21 and bone density remains controversial.^77,78^

### Cell level

#### Telomeres

**Fig. 4:**
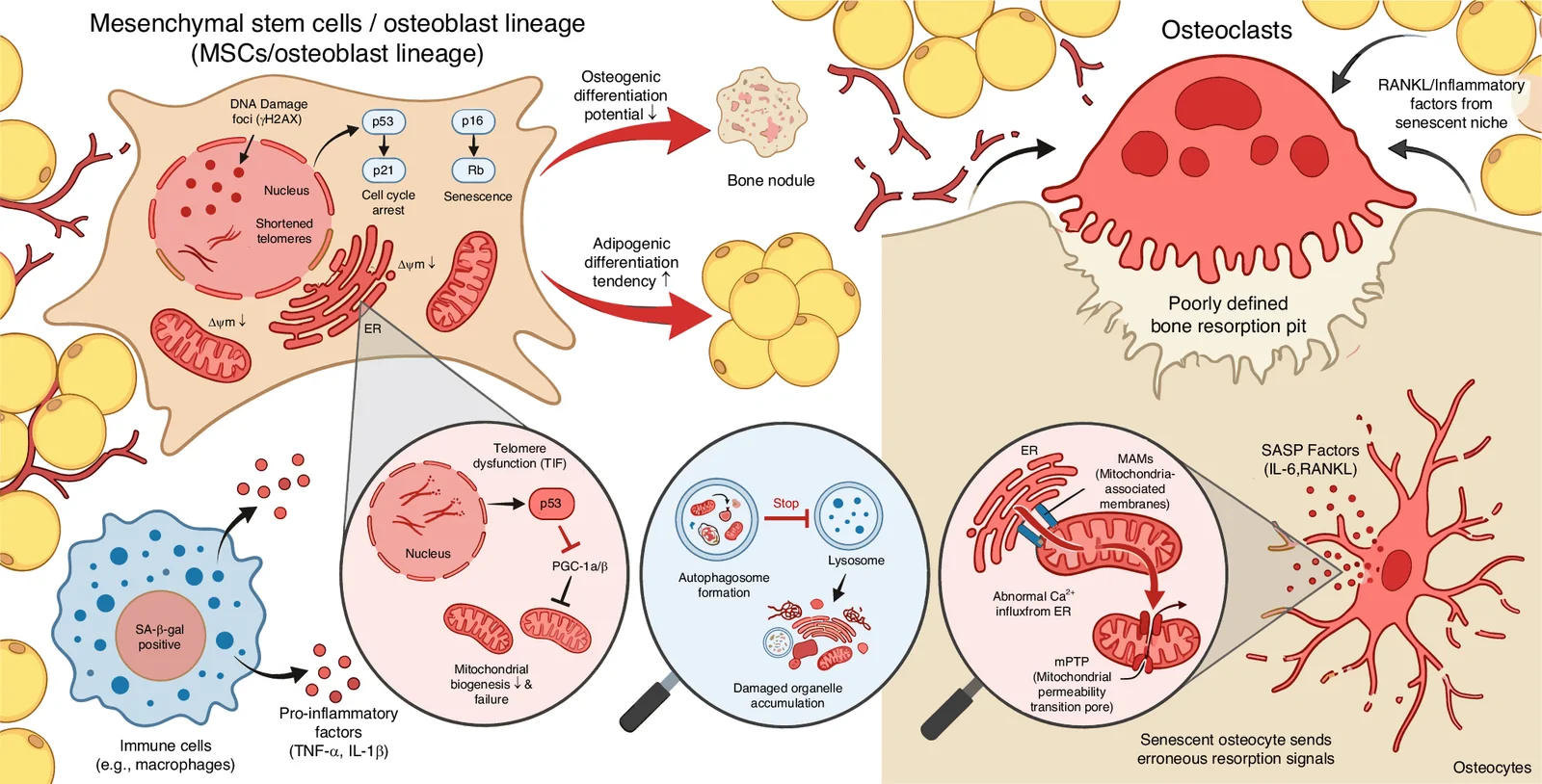
Cellular senescence in the skeletal niche. This figure focuses on the aging status of key cell populations and their subcellular lesions in the bone microenvironment. In the context of an aging bone marrow with increased steatosis and decreased vascularity, four types of cells are highlighted: mesenchymal stem cells/osteoblasts, osteoclasts, osteocytes, and immune cells. The close-up of three magnmagnies further reveals common molecular lesions such as telomere-mitochondrial axis disorders, autophagic flow disorders, and ER-mitochondrial calcium homeostasis imbalance, which jointly drive decreased bone formation and increased bone resorption

Telomeres are protective nucleoprotein structures whose progressive shortening is a core hallmark of cellular senescence. Within the skeletal system, dysregulation of telomere homeostasis is considered a significant cellular and molecular mechanism driving bone aging, although existing evidence remains heterogeneous (Fig. 4).

Several observational studies have reported positive associations between leukocyte telomere length and BMD or reduced fracture risk,^79,80^ but an approximately equal number of well-powered studies—including a regression analysis of 2 750 community-dwelling older adults,^81^ a cohort of 1 867 older Chinese adults,^82^ and UK Biobank data^83^—have found no association with BMD, osteoporosis, fracture risk, or other skeletal parameters. This inconsistency cannot be dismissed as mere sampling variation; rather, it suggests that the telomere–bone relationship, if real, is likely modest in effect size and heavily modulated by population characteristics, skeletal site, and unmeasured confounders such as chronic infection or inflammation.^84^ Mendelian randomization analyses have further failed to support a causal link between telomere length and sarcopenia,^85^ raising the possibility that observed associations in observational studies reflect confounding rather than causality.

Experimental models provide mechanistic evidence that telomere dysfunction can drive bone aging under certain conditions—radiation-induced models show telomere dysfunction-induced foci (TIFs) in osteocytes,^34^ and genetically engineered mice with disruptions in telomere maintenance genes (Wrn, Terc) exhibit impaired osteoblast differentiation and osteoporosis.^86,87^ However, the translational relevance of these models is questionable: radiation-induced damage and complete gene knockouts represent extreme perturbations that differ substantially from the gradual, physiological telomere attrition that occurs in human aging. Whether the TIFs and senescence markers observed in these models appear in normally aging human bone—and if so, at what frequency—remains largely unknown. Thus, while telomere dysfunction can cause bone pathology in experimental settings, its contribution to typical human skeletal aging remains unproven, and the field would benefit from prospective studies that directly measure telomere dynamics in bone tissue rather than relying on leukocyte proxies or extreme genetic models.

#### Mitochondria

Mitochondria function as cellular powerhouses and play a dual role in maintaining skeletal homeostasis. They generate energy via oxidative phosphorylation while also producing ROS as crucial signaling molecules. To fully elucidate how mitochondrial dysfunction drives bone aging, three interconnected mechanistic pathways should be distinguished, along with the unresolved controversies.

First, the ROS-DDR-p53/p21 axis represents the most classical pathway. Excessive mitochondrial ROS directly induce DNA double-strand breaks, activating the DNA damage response and downstream p53/p21-mediated cell cycle arrest.^88^ However, emerging evidence challenges the unidirectional model: moderate ROS elevation can paradoxically promote osteoblast differentiation via Nrf2-mediated antioxidant gene activation, suggesting a hormetic dose-response relationship that shifts from beneficial to deleterious with age.^89^ Moreover, whether ROS acts primarily as an initiator or a perpetuator of senescence remains debated, as senescent cells themselves produce ROS, creating a self-reinforcing loop.

Second, the mitophagy-mitochondrial dynamics axis garnered increasing attention. While impaired mitophagy clearly accelerates bone aging—as evidenced by PINK1 deficiency leading to excessive mitochondrial fusion and senescent cell accumulation—the role of mitochondrial fragmentation versus hyperfusion is contested.^90^ Some studies report that Drp1-mediated fragmentation promotes senescence,^91^ whereas others demonstrate that elongated, hyperfused mitochondria are characteristic of aged osteoblasts and protect against apoptosis.^92^ This discrepancy may reflect cell-type specificity or the duration of stress exposure, with transient fragmentation facilitating mitophagy but chronic fragmentation overwhelming clearance capacity.

Third, the mitochondrial-ER crosstalk via mPTP and calcium signaling has emerged as a critical but understudied mechanism. The recent discovery that STING promotes mPTP opening through the HK2-VDAC1 axis provides a molecular link between innate immunity and mitochondrial dysfunction in bone aging.^93^ Nevertheless, divergent viewpoints exist regarding the temporal order of events: some argue that mPTP opening is a late-stage consequence of severe mitochondrial damage, while others posit it as an early trigger of senescence.

#### Endoplasmic reticulum

The endoplasmic reticulum (ER) is a crucial organelle responsible for protein synthesis, folding, modification, and calcium storage. Disruption of ER homeostasis, commonly referred to as ER stress, is a significant mechanism underlying cellular senescence and skeletal degeneration. Functional defects in ER function can directly lead to bone disease; for instance, mutations in TMEM38B disrupt ER calcium homeostasis, impair type I collagen folding and secretion, and result in osteogenesis imperfecta.^94–96^ In bone aging, ER stress exerts a biphasic effect on skeletal homeostasis. Moderate or transient ER stress may promote osteogenesis, whereas prolonged or excessive stress shifts cellular responses toward catabolism.^97^ For instance, mild ER stress induced by curcumin enhances osteogenic differentiation of mesenchymal stem cells via activation of the ATF6 pathway,^98^ whereas intracellular accumulation of AGEs induces severe ER stress, leading to osteoblast apoptosis.^99^

Multiple pathological stimuli associated with aging can trigger ER stress in bone. Glucocorticoids inhibit bone marrow stem cell proliferation and induce osteoblast apoptosis by activating the unfolded protein response, particularly the PERK-NRF2 pathway, which is a central mechanism underlying glucocorticoid-induced osteoporosis. Pharmacological inhibition of ER stress with 4-phenylbutyric acid mitigates this apoptotic effect. Long-term use of proton pump inhibitors and the immunosuppressant cyclosporine A has also been linked to increased osteoporosis risk through ER stress activation and ROS accumulation.^100,101^ Metabolic stressors such as the saturated fatty acid palmitate induce lipid droplet accumulation and senescence in nucleus pulposus cells via ER stress pathways.^102^ Furthermore, ER stress impairs myogenic responses to anabolic stimuli and alters bone cell mechanosensitivity, thereby contributing to both sarcopenia and osteopenia.^103^ In intervertebral disc degeneration, deficiency of site-1 protease disrupts protein transport, leading to ER dilation, abnormal ER-mitochondrial calcium flux, and ultimately mitochondrial dysfunction and a senescent phenotype.^104^

ER stress further drives skeletal catabolism and inflammation through activation of downstream signaling pathways. Studies indicate it promotes inflammation, apoptosis, and autophagy via the p38 MAPK signaling pathway, leading to bone loss.^105^ In BMSCs, ER stress enhances inflammation-induced bone loss by activating the ROCK1 signaling.^106^ Additionally, specific non-coding RNAs are involved in ER stress regulation; for example, miR-1260b targets ATF6β to modulate ER stress and attenuate periodontal bone loss.^107^

#### Proteostasis

Protein homeostasis, or proteostasis, is fundamental to the maintenance of bone cell function. In the context of bone aging, failure of proteostasis directly impairs three critical processes: the secretory capacity of osteoblasts for type I collagen and other matrix proteins; the survival and mechanosensing function of long-lived osteocytes; and the tightly regulated resorptive activity of osteoclasts. Consequently, age-related decline in proteostasis is not merely a general cellular phenomenon but a specific driver of skeletal degeneration.

For the chaperone system (HSP70/HSP90), in aged bone, the imbalance between HSP70 and HSP90 tips the osteoblast-osteoclast coupling toward resorption, as HSP70 can promote osteoclastogenesis by activating the RANKL pathway via TLR signaling^108^ by cooperating with macrophage colony-stimulating factor (M-CSF) to induce transdifferentiation of dendritic cells into osteoclasts.^109^ In contrast, HSP90 negatively regulates RANKL expression and osteoclast formation.^110^

For the ubiquitin-proteasome system, age-related decline in proteasome function in osteoclast precursors leads to the accumulation of pro-resorptive signaling molecules, exacerbating bone loss.^111^ Accordingly, proteasome inhibitors such as bortezomib and carfilzomib can reduce bone resorption by disrupting key signaling pathways involving p62 and TRAF6, offering potential therapeutic strategies for osteoporosis.^112^ Additionally, gasdermin D (GSDMD) has been identified as a lysosomal checkpoint protein with anti-osteoclast activity that protects against bone loss.^113^

For the autophagy-lysosome system, in aged osteocytes, autophagic failure is particularly detrimental, leading to the accumulation of damaged organelles, oxidative stress, and senescence. AMPK promotes osteoblast differentiation and mineralization by inducing autophagy^114^ and protects osteoblasts from apoptosis by clearing damaged organelles and limiting ROS accumulation.^115^ Age-related decline in autophagic capacity, particularly in long-lived osteocytes, leads to intracellular waste accumulation and a senescent phenotype, thereby disrupting bone metabolic balance.

#### Deregulated nutrient sensing

Deregulated nutrient sensing during aging reflects impaired cellular capacity to detect and respond appropriately to nutritional signals such as glucose and amino acids. This dysfunction represents a core mechanism linking systemic metabolic abnormalities to skeletal degeneration and is driven primarily by an imbalance among the insulin/IGF-1, mTORC1, AMPK, and sirtuin signaling pathways, which collectively reshape the metabolic microenvironment of the aging skeleton.^116^

The insulin/IGF-1 signaling pathway is a potent anabolic signal in bone. As an important target organ for insulin, bone exhibits features of insulin resistance during aging and in metabolic bone disorders.^117^ Activation of this pathway promotes osteoblast proliferation, differentiation, and survival via the PI3K/AKT signaling pathway, whereas insulin resistance blunts osteoblast anabolic responses. Impaired osteoblast function also disrupts the production and activation of osteocalcin, a hormone that regulates systemic insulin sensitivity. This dysregulation establishes a vicious cycle within the bone-pancreas axis, exacerbating both local skeletal and systemic metabolic disturbances.^118^

The mTORC1 pathway integrates growth factor, energy, and amino acid signals and undergoes significant functional changes with aging.^119^ While physiological mTORC1 activity supports bone formation, chronic overactivation during aging has deleterious consequences. It induces premature senescence in osteoprogenitor cells, suppresses their differentiation, and inhibits autophagy, leading to the accumulation of toxic intracellular substances, impaired osteoblast function, and increased apoptosis.^120^ Furthermore, mTORC1 promotes osteoclast differentiation and survival, and its excessive activation accelerates bone resorption. Genetic disruption of negative mTORC1 regulators in osteoclasts results in severe age-related bone loss.^121^

AMPK functions as a central cellular energy sensor and generally antagonizes mTORC1 signaling. Its activity declines with age,^122^ leading to multiple adverse effects. Reduced AMPK signaling shifts osteoblast metabolism from efficient oxidative phosphorylation toward glycolysis, insufficiently supporting the energy demands of bone formation. As a crucial initiator of autophagy, AMPK deficiency impairs cellular clearance functions, accelerating organelle damage and functional deterioration. Furthermore, diminished AMPK activity weakens antioxidant defenses mediated by FOXO, increasing susceptibility to oxidative stress and accelerating senescence.^123^

The sirtuin family of NAD⁺-dependent deacetylases, particularly SIRT1, SIRT3, and SIRT6,^124^ plays a crucial role in bone aging. Age-related declines in NAD⁺ levels reduce sirtuin activity. Loss of SIRT1 impairs osteogenic differentiation and favors adipogenesis; deficiency of the mitochondrial SIRT3 leads to mitochondrial dysfunction and excessive ROS production in osteoblasts; and SIRT6 loss disrupts osteoblast metabolism and genomic stability, producing a premature aging-like skeletal phenotype.^125^

#### Cellular senescence

Cellular senescence is a state of irreversible cell cycle arrest accompanied by specific phenotypic alterations.^126–128^ It represents a fundamental mechanism driving the degeneration and functional decline of bone tissue.^129^ Multiple cell types within bone accumulate senescent cells with advancing age.

Senescence of the osteoblastic lineage. Senescence of osteoblasts and their progenitors, including osteoprogenitor cells and MSCs, directly contributes to reduced bone formation. Due to replicative senescence and telomere attrition, the limited proliferative capacity of MSCs and osteoprogenitors leads to progressive telomere shortening, ultimately triggering the DDR-p53/p21CIP1-p16INK4a/Rb pathways, inducing cell cycle arrest.^130^ Senescent osteoblasts exhibit significantly reduced proliferation, differentiation capacity, and mineralized nodule formation.^131^

Senescence of osteoclasts. Osteoclasts originate from hematopoietic stem cells, and research on their senescence is relatively limited. Nevertheless, evidence suggests that the senescent bone marrow microenvironment affects the differentiation and function of osteoclast precursors. Senescence-associated inflammatory factors may abnormally stimulate osteoclastogenesis, and senescent osteoclasts themselves may exhibit dysregulated resorptive activity.^132^

Senescence of osteocytes. Osteocytes are the longest-lived cells in the bone matrix and serve as sensors for mechanical stress and regulators of bone remodeling. Aging osteocytes undergo network degradation and increased apoptosis but also acquire a senescent phenotype. Altered SASP profiles in senescent osteocytes may transmit aberrant pro-resorptive signals to osteoclast precursors while inhibiting osteoblast activity, thereby disrupting bone homeostasis.^5^

Senescence of BMSCs. BMSCs are common precursors to osteoblasts and adipocytes. Senescent BMSCs exhibit reduced osteogenic potential and a bias toward adipogenic differentiation, leading to increased marrow adipose tissue (marrow adiposity). This shift further compromises the hematopoietic and osteogenic microenvironment.^133^

Vascular senescence in bone. Angiogenesis is tightly coupled to osteogenesis. With aging, bone endothelial cells (BECs) acquire a senescent phenotype, characterized by telomere shortening and increased p16INK4a expression.^134^ Senescent BECs exhibit impaired capacity to form H-type vessels, which are specialized capillaries that couple angiogenesis with osteogenesis by secreting factors such as SLIT3 and RANKL antagonists.^135^ Age-related loss of H-type vessels directly reduces perivascular osteoprogenitor cells and impairs bone formation.^135^

Neural senescence in bone. In the peripheral nervous system, sympathetic nerve density in bone decreases with age, while the remaining nerves exhibit signs of dysfunction, including reduced norepinephrine reuptake capacity and altered neuropeptide Y expression.^136^ This dysregulation leads to an unbalanced sympathetic tone, promoting osteoclast activity via β2-adrenergic receptors on osteoblasts and osteoclast precursors. Sensory nerves, which release calcitonin gene-related peptide (CGRP) and substance P with pro-osteogenic and vasodilatory effects, also show age-dependent degeneration.^137^

### Microenvironment level

#### Chronic inflammation

**Fig. 5:**
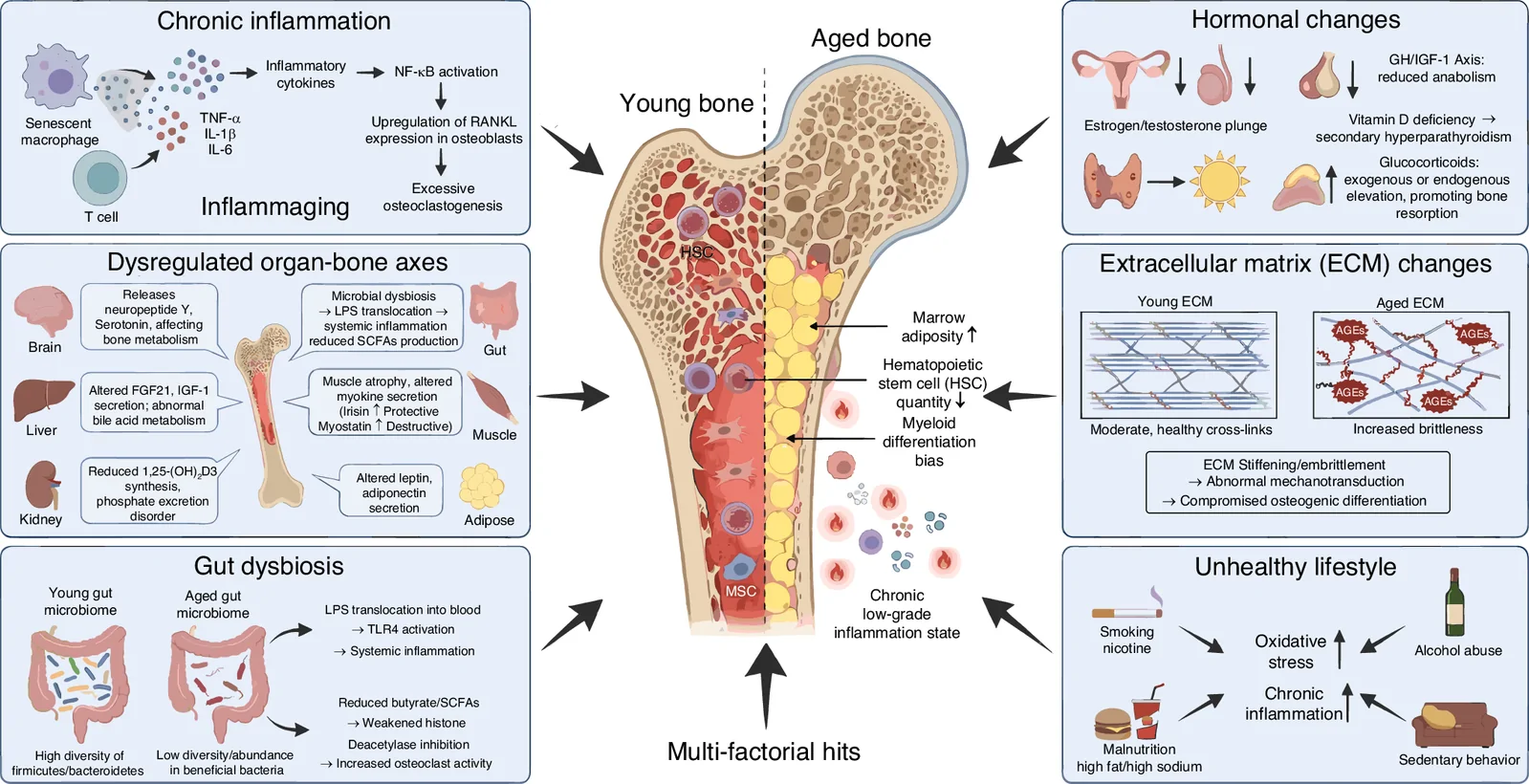
Systemic drivers and microenvironmental disruption in bone aging. This figure illustrates the multi-system drivers of bone aging and their synergistic disruption of the local bone marrow microenvironment. The center of the figure shows the characteristics of aging bones: fatty red bone marrow, depletion of hematopoietic stem cells, and chronic low-grade inflammation. It is surrounded by six driver modules, including: 1) osteogenesis activated by chronic inflammation; 2) multiple hormone axis changes; 3) multiple organ-bone axis disorders; 4) extracellular matrix hardening and fragility; 5) dysbiosis of gut microbiota leading to translocation of endotoxin and reduction of beneficial metabolites; 6) unhealthy lifestyles increase oxidative stress and inflammation. All systemic blows eventually converge and disrupt local bone homeostasis

Chronic low-grade inflammation is a fundamental and persistent feature of bone aging (Fig. 5). The senescence of hematopoietic stem cells (HSCs) constitutes a central component of immunosenescence, and the aging bone marrow microenvironment plays a pivotal role in driving this process. With advancing age, red bone marrow is progressively replaced by adipose tissue, resulting in diminished nutritional and regulatory support. Concurrently, the accumulation of senescent cells and pro-inflammatory mediators collectively impairs HSC function.^138,139^ Under inflammatory stress, HSC metabolism shifts from glycolysis toward oxidative respiration. The accumulated ROS can trigger DNA damage, senescence, or apoptosis.^140^ The CXCR4/CXCL12 signaling axis has been shown to protect HSCs by attenuating mitochondrial oxidative stress.^108^ Notably, the transcription factor Bcl11a exhibits a dual role: while exerting cell-intrinsic protective effects, it simultaneously promotes bone marrow inflammation, thereby exacerbating HSC dysfunction.^141^ Inflammation also induces a myeloid bias in HSC differentiation, favoring myeloid over lymphoid lineage commitment and contributing to immune imbalance. Interestingly, skull bone marrow appears more resistant to this aging-related shift than femoral bone marrow.^142^

Senescent immune cells and the pro-inflammatory factors they secrete are the direct executors disrupting bone homeostasis. Aging bone marrow exhibits increased numbers and activity of senescent macrophages, T lymphocytes, and other immune cells, resulting in sustained secretion of factors such as TNF-α, IL-1β, and IL-6.^143,144^ These cytokines are potent stimulators of osteoclast differentiation: through activation of NF-κB and MAPK signaling pathways, they upregulate RANKL expression in osteoblasts and activated T cells, thereby promoting excessive osteoclastogenesis, prolonged lifespan, and hyperactive bone resorption.^145^ Senescent skeletal stem cells further contribute to the formation of an inflammatory degenerative niche, characterized by diminished bone formation potential and enhanced ability to secrete pro-inflammatory and pro-resorptive cytokines, further skewing normal differentiation within the skeletal and hematopoietic systems.^146^ Additionally, senescent fibroblasts exacerbate inflammation and osteoclast differentiation via activation of mTOR signaling,^147^ while aging disrupts the normal circadian regulation of macrophage function.^148^

This chronic inflammatory state ultimately leads to bone loss and increased skeletal fragility through multiple intersecting mechanisms. Pro-inflammatory cytokines not only directly stimulate bone resorption but also impair bone formation by inducing osteoblast apoptosis and pyroptosis and by disrupting autophagic processes, thereby disrupting the osteoblast-osteoclast coupling.^143–145^ Interventions targeting this inflammatory axis show therapeutic promise. For instance, IL-11 inhibitors have been shown to improve metabolic and muscle function by modulating the ERK-AMPK-mTORC1 axis and extend the lifespan in experimental models.^149^

#### Hormone level changes

Age-related hormone alterations represent one of the core intrinsic factors driving bone aging. Bone homeostasis is regulated by a complex network, and age-associated declines, dysregulation, and altered interactions among hormones collectively create an endocrine aging environment leading to skeletal homeostatic imbalance.

The decline in sex hormones is among the most impactful events. In women, the abrupt reduction in estrogen levels during menopause directly enhances osteoclast activity, increases osteoblast apoptosis, reduces bone mechanosensitivity, and intensifies local inflammation, leading to rapid early postmenopausal bone loss.^150^ In contrast, men experience a gradual decline in gonadal function. Reduced testosterone not only diminishes androgen receptor-mediated anabolic signaling in bone but also decreases its aromatization to estrogen, resulting in a combined androgen and estrogen deficiency that is closely associated with age-related bone loss in older men.^151^

The growth hormone/IGF-1 axis also progressively attenuates with age. This axis is critical for the acquisition of peak bone mass during adolescence, and its decline leads to reduced proliferation and differentiation of osteoblast precursors, lower bone formation rates, impaired bone matrix quality, and exacerbation of sarcopenia, thereby reinforcing the vicious cycle of sarcopenia-osteoporosis.^152^

Dysregulation of the parathyroid hormone (PTH) and vitamin D systems often occurs in parallel during aging. Vitamin D deficiency reduces intestinal calcium absorption, triggering a compensatory increase in PTH levels.^153^ Chronic mild elevations in PTH promote cortical bone resorption, while vitamin D deficiency also directly further compromises osteoblast function and mineralization.^154^

Additional hormonal changes also contribute significantly. Declines in endogenous calcitonin secretion may weaken its inhibitory effects on osteoclast activity.^155^ The widespread use of exogenous glucocorticoids remains a major iatrogenic cause of secondary osteoporosis in older adults.^156^ Thyroid dysfunction accelerates bone turnover, leading to net bone loss.^157^ Furthermore, age-related changes in adipose tissue distribution alter the secretion of adipokines such as leptin and adiponectin, which influence bone metabolism in complex and context-dependent manners.^158,159^ Hormonal changes in bone aging are not isolated events but rather an interconnected network. This network-based dysregulation may explain the limited efficacy of interventions targeting single hormonal pathways.

#### Extracellular matrix changes

The bone extracellular matrix (ECM) is not merely a structural scaffold but an active regulator of cell fate, signaling, and tissue mechanics. Dynamic alterations in ECM composition during aging represent a central mechanism driving bone aging.^160^

With aging, ECM components undergo progressive degradation, directly impairing bone quality and function. The deterioration of the collagen network is particularly critical: type I collagen synthesis declines, while degradation increases, leading to net loss. Moreover, collagen cross-linking shifts from physiological enzymatic cross-links to pathologic AGE-mediated cross-links, rendering collagen fibers brittle and inflexible and forming a structural basis for increased bone fragility.^161^ Concurrently, collagen fiber alignment becomes disorganized, and the expression and modification of non-collagenous proteins that influence hydration and signaling, such as osteocalcin, osteopontin, and glycosaminoglycans, are dysregulated.^162^ These changes impair ECM mineralization control, growth factor storage, and cell-matrix communication.

Altered ECM mechanics profoundly affect cellular mechanosensing. At the macroscopic level, aging ECM exhibits reduced elasticity and increased brittleness.^160^ At the nanoscale, changes in stiffness and elasticity transmit dysregulated mechanical signals to cells via adhesion molecules such as integrins. This disruption of mechanotransduction impairs osteogenic differentiation, skewing mesenchymal stem cells toward adipogenic differentiation and also causing osteocyte dysfunction.^163^ As the core mechanosensors in bone, the lacunar-canalicular network of osteocytes becomes distorted and obstructed due to pathological changes in the ECM, reducing the efficiency of their ability to sense and transmit mechanical signals. This, in turn, leads to an imbalance in their secretion of key regulatory factors like sclerostin and RANKL, exacerbating bone resorption.^164^

As a regulatory hub for cellular signaling, ECM aging also disrupts key biological signal transduction. The ECM serves as a natural reservoir for pro-osteogenic growth factors such as TGF-β, BMPs, and IGF-1. Changes in its composition and structure affect binding, presentation, and release of these factors, potentially leading to their abnormal retention or degradation, thereby weakening osteogenic signals.^165^ Furthermore, osteoblasts and osteocytes bind to the ECM via integrins. Alterations in ligands within the aging ECM result in insufficient activation of integrin-mediated downstream signaling, subsequently impairing cell survival, proliferation, and differentiation capacities.^166^

## Intervention and treatment

### Non-pharmacological interventions

**Fig. 6:**
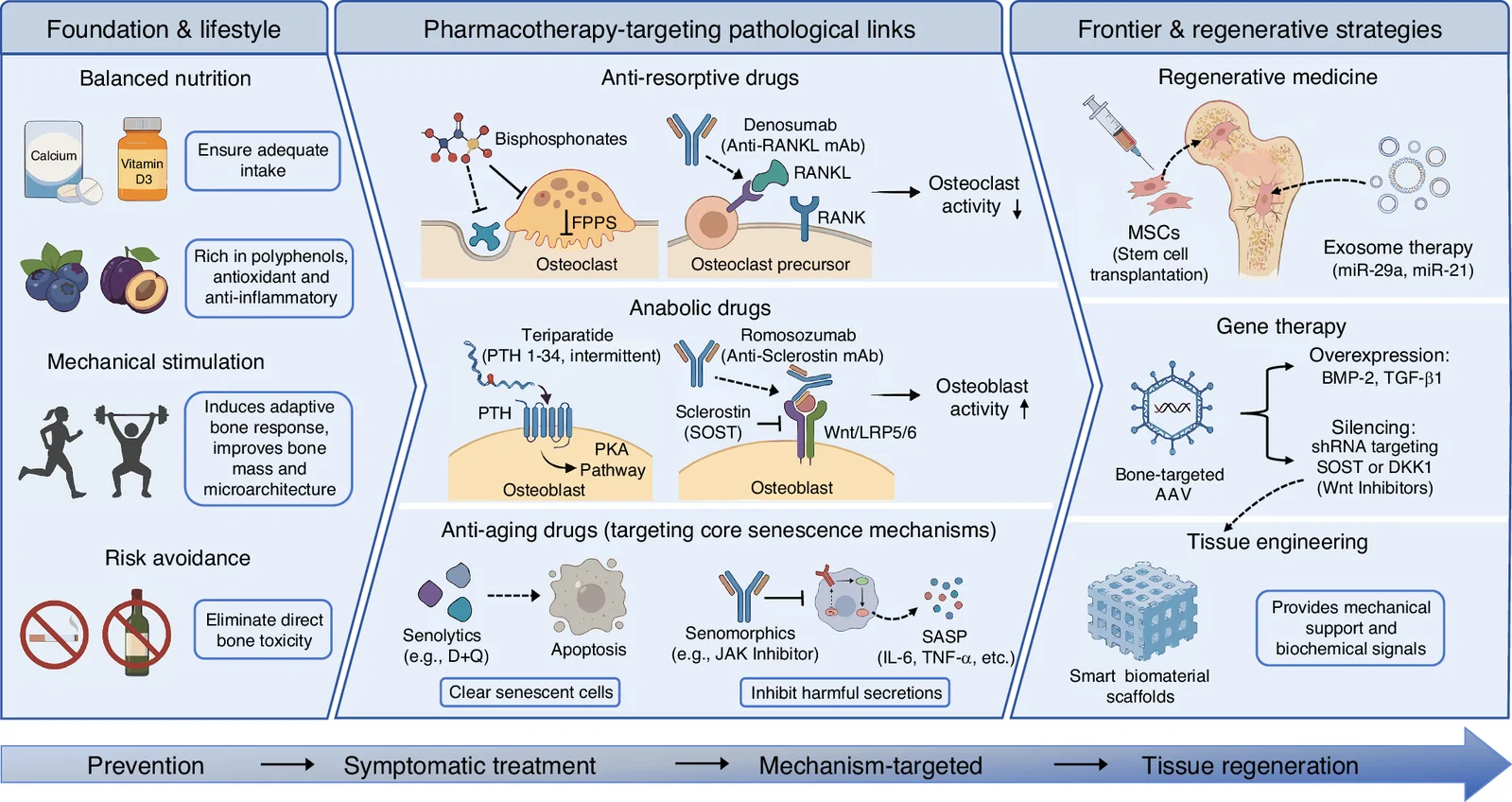
Therapeutic strategies targeting bone aging at multiple levels. This figure systematically shows the multi-level therapeutic strategies for targeted bone aging, and the layout reflects the evolution of intervention concepts from the basic to the cutting-edge. The left column is the basic lifestyle intervention, emphasizing the central role of balanced nutrition and mechanical stimulation (see Fig. 5 for pathological mechanisms). In the middle column, drugs targeting the pathological aspects of aging, including anti-resorptive drugs, anabolic drugs, and anti-aging drugs targeting the core mechanisms of aging. The right column shows cutting-edge regenerative strategies, including stem cell and exosome therapy, bone-targeted gene therapy, and tissue engineering. The bottom timeline summarizes the strategic development path from prevention and symptomatic treatment to targeted mechanisms and tissue regeneration

Lifestyle interventions are the cornerstone of bone health across the lifespan. However, their effectiveness depends critically on timing, dose, adherence, and individual baseline characteristics. Three principles guide their implementation: (1) primary prevention should begin early (childhood to young adulthood) to maximize peak bone mass; (2) secondary prevention in older adults focuses on slowing bone loss and reducing fall risk; (3) interventions show dose-response relationships, and “more is not always better”—excessive exercise or calcium supplementation may have diminishing returns or adverse effects. The following subsections provide specific, quantifiable recommendations and, where evidence permits, comparative efficacy estimates (Fig. 6).

#### Balanced diet

A balanced intake of macronutrients is fundamental to skeletal health. Adequate, but not excessive, protein consumption is essential for the synthesis of bone matrix collagen; insufficient protein intake compromises both muscle and bone integrity. The quality and balance of dietary fatty acids are equally crucial. Increased consumption of foods rich in omega-3 polyunsaturated fatty acids helps mitigate inflammation and inhibit osteoclast activity, whereas intake of saturated and trans fats should be reduced.^167^ Sufficient calcium and vitamin D intake are direct prerequisites for preventing accelerated bone aging, requiring special attention for supplementation in older adults and postmenopausal women.^168^ However, the latest evidence published in the *BMJ* indicates that calcium, vitamin D, or combined supplements offer little or no benefit in preventing fractures and falls.^169^ Vitamin K, which is required for the activation of proteins such as osteocalcin that facilitate appropriate calcium deposition in bone, can be obtained from leafy green vegetables and fermented foods.^170^ Furthermore, micronutrients such as magnesium, potassium, zinc, and vitamin C are extensively involved in bone metabolism and matrix synthesis, and their deficiencies can indirectly harm skeletal health.^171^

The regulatory role of dietary phytochemicals in bone health is increasingly recognized. Polyphenols, carotenoids, and other compounds abundant in fruits and vegetables possess potent antioxidant and anti-inflammatory activities, protecting bone by inhibiting osteoclastogenesis and promoting osteogenic signaling pathways. Evidence from research on specific berries supports these effects. Early-life consumption of blueberries has been shown to prevent osteoblast aging and bone loss in adulthood,^172,173^ while plums, sour cherries, bilberries, and other fruits have demonstrated efficacy in ameliorating age-related bone loss.^174–180^ These benefits may be mediated through modulation of the gut-bone axis and antioxidant mechanisms.^181^ Population-based studies further indicate that higher fruit intake during childhood is associated with greater femoral neck BMD in adulthood,^182^ and that dietary patterns rich in fruits, vegetables, and whole grains among middle-aged and older adults can reduce fracture risk (female HR: 0.86, 95% CI 0.76-0.98, male HR: 0.83, 95% CI 0.64-1.08).^183^

Based on current evidence,^184^ the following daily intakes are recommended for older adults: calcium 1 000–1 200 mg/day (dietary priority over supplements; 500–600 mg per meal maximizes absorption); vitamin D 800–1 000 IU/day (higher doses, e.g., >2 000 IU/day, show no additional bone benefit^185^); protein: 1.0–1.2 g/kg body weight/day (higher, 1.2–1.5 g/kg, for frail older adults with sarcopenia). vitamin K (90–120 μg/day) from leafy green vegetables supports osteocalcin activation, while magnesium (320–420 mg/day) and zinc (8–11 mg/day) are cofactors for bone metabolism. Among dietary patterns, the Mediterranean diet (rich in fruits, vegetables, whole grains, and olive oil) shows the strongest evidence for fracture prevention, compared to calorie restriction (pooled hazard ratios = 0.95, 95% CI 0.93-0.96), low-carbohydrate/ketogenic diets, and higher-protein diets.^186^

#### Moderate exercise

Regular physical activity is an effective non-pharmacological intervention for maintaining skeletal health and combating bone aging. The benefits of exercise include increases in BMD, improvements in bone quality, and reductions in fracture risk. The effects of exercise on BMD are modality-dependent.^187^ Weight-bearing exercises and resistance training are particularly effective. Weight-bearing exercises stimulate bone through ground reaction forces, whereas resistance training imposes tensile loads through muscle contraction, both of which elicit adaptive responses in bone. Regular engagement in moderate- to high-intensity forms of these activities can effectively slow age-related BMD loss at clinically important sites such as the lumbar spine and femoral neck, with particularly pronounced benefits observed in postmenopausal women.^188^

Beyond BMD, exercise enhances bone quality by improving trabecular microarchitecture and increasing geometric parameters such as cross-sectional area, thereby strengthening resistance to bending and torsion.^189^ In older adults with established osteoporosis, supervised, individualized, and progressive intensified strength and balance training, although potentially producing only modest increases in BMD, plays a critical role in improving muscle strength, coordination, and postural stability. These functional gains substantially reduce the risk of falls and, consequently, fragility fractures.^190^

For BMD gains, the following exercise prescriptions are recommended based on meta-analytic evidence^191^: resistance training at 60%–80% of one-repetition maximum, 8–12 repetitions × 2–3 sets, 2–3 sessions per week; weight-bearing aerobic exercise (walking, jogging, stair climbing) for 30–45 minutes per session, 3–5 sessions per week; combined (multicomponent) training incorporating both resistance and weight-bearing elements. For fall prevention, balance training (tai chi, standing exercises, dynamic balance tasks) should be performed at least 2 sessions per week. For frail or deconditioned older adults, a “start low, go slow” approach is recommended, with intensity progression every 4–6 weeks as tolerated. High-impact exercise (jumping, hopping) produces larger BMD gains but is contraindicated in individuals with established osteoporosis or high fracture risk.

Comparative efficacy: A meta-analysis of 74 RCTs (*n* = 5 112) compared the effects of different exercise modalities on lumbar spine BMD in postmenopausal women: combined training > resistance training > weight-bearing aerobic, with effect sizes (SMD) of 0.42, 0.40, and 0.26, respectively.^192^ For fall prevention, a 2025 meta-analysis found Multicomponent physical activity (combining strength, balance, and aerobic training) and mind-body exercises (tai chi, yoga) demonstrated the most consistent benefits.^193^ Regarding the effect of different training frequency, Zitzmann et al. found that training at least twice per week has a significantly greater impact on lumbar BMD compared to a lower frequency (<2 times per week) (SMD = 0.55, 95% CI = 0.20-0.90).^194^

#### Restriction of alcohol and tobacco use

Tobacco use and excessive alcohol intake are independent yet synergistic risk factors for bone health. Smoking adversely affects bone health through direct cellular toxicity, disruption of sex hormone balance, induction of oxidative stress and inflammation, impairment of vascular function, and interference with vitamin D and calcium metabolism.^195,196^ Excessive alcohol intake further compromises skeletal integrity by suppressing osteoblast activity, promoting secondary osteoporosis, increasing fall risk, and delaying fracture healing.^197–199^

For adolescents and young adults who have not yet achieved peak bone mass, abstaining from smoking and limiting alcohol intake are essential for building maximal skeletal reserve. In perimenopausal women and middle-aged men, these measures represent a first-line defense against age-related physiological bone loss. In individuals with osteoporosis, continued smoking and alcohol consumption can reduce the therapeutic efficacy of anti-osteoporotic medications such as bisphosphonates, denosumab, and teriparatide.^200–202^ Nicotine and alcohol interfere with the microenvironment of the target cells of these agents, potentially leading to a suboptimal therapeutic response. Therefore, smoking cessation and moderation of alcohol intake are essential prerequisites to ensure that pharmacological treatments achieve their intended effects.

### Pharmacotherapy

#### Antiresorptive agents

Bisphosphonates: Oral bisphosphonates, such as alendronate, risedronate, and zoledronate, remain the most cost-effective interventions. They selectively bind to the mineralized bone matrix and are internalized by osteoclasts, where they disrupt the mevalonate pathway, inducing apoptosis and thereby potently inhibiting bone resorption.^203^ Long-term studies have demonstrated significant reductions in vertebral and hip fracture risk. However, prolonged use requires careful monitoring because of rare but serious adverse events, including osteonecrosis of the jaw and atypical femoral fractures.^204^ Contemporary real-world data from large cohort studies (*n* > 26 000) have refined the risk estimates: atypical femoral fractures occur at a rate of approximately 1.93 per 1 000 person-years after three years of continuous use, rising to 2.50 per 1 000 person-years after five years, whereas osteonecrosis of the jaw remains exceedingly rare at less than 0.07 per 1 000 person-years^205^ (Table 1).

**Table 1:** Pharmacotherapy for bone aging

Drug Category		Representative Drugs	Mechanism	Indications	Adverse Reactions	Special Considerations
Antiresorptive Agents	Bisphosphonates	Alendronate	farnesyl pyrophosphate synthase inhibition → osteoclast apoptosis	First-line for postmenopausal OP; male OP; glucocorticoid-induced OP; Paget’ s disease	AFF; ONJ; Esophagitis	Drug holiday after 3–5 years; most cost-effective
		Zoledronate				Convenient once-yearly; preferred after hip fracture
		Risedronate				Faster onset than alendronate
	RANKL Monoclonal Antibody	Denosumab	RANKL neutralization	Postmenopausal OP; male OP; glucocorticoid-induced OP	ONJ; AFF; rebound vertebral fractures; hypocalcemia	No drug holiday; never discontinue without transition to bisphosphonate; rebound bone loss within 6 months
	Selective Estrogen Receptor Modulators	Raloxifene	Estrogen agonist in bone	Postmenopausal OP; prevention in women with high invasive breast cancer risk	VTE; hot flashes; leg cramps	No hip fracture benefit; favorable breast safety profile
	Hormone Replacement Therapy	Estradiol	estrogen receptor on osteoblasts → reduces osteoclast activity	Younger postmenopausal women (age ≤60 yrs) with menopausal symptoms and high fracture risk	Stroke; VTE; breast cancer (long-term combined therapy)	Not recommended for women >60 yrs or without menopausal symptoms; reserve for those with moderate-to-severe vasomotor symptoms
Anabolic Agents	PTH/PTHrp Analogs	Teriparatide	PTH1R activation	Very high fracture risk	Hypercalcemia; leg cramps; dizziness	Max 24 months lifetime; must follow with anti-resorptive
		Abaloparatide	PTH1R activation (selective)			
	Sclerostin Monoclonal Antibody	Romosozumab	Sclerostin inhibition → Wnt activation		CV events; ONJ; AFF	Max 12 months; must follow with anti-resorptive; assess CV risk before use
Senolytic Therapies	Senolytic Agents	Dasatinib/ Quercetin	BCL-2/HSP90/SCF inhibition → selective senescent cell apoptosis	Investigational for age-related bone loss; postmenopausal OP	Generally well-tolerated	High senescence burden subgroup showed BMD gains; biomarker-driven therapy
	SASP Modulators	Ruxolitinib	JAK-STAT inhibition → suppression of SASP	Investigational for age-related bone loss; preclinical models	To be determined	Not yet tested in clinical trials for bone
		Rapamycin	mTOR inhibition → suppression of SASP		To be determined	Known immunosuppressive effects limit long-term use
		Fisetin	Multiple pathways		Generally recognized as safe (food-derived)	Natural flavonoid; senolytic activity demonstrated in preclinical models; human bone data lacking

*AFF* atypical femoral fracture, *ONJ* osteonecrosis of the jaw, *VTE* venous thromboembolism, *CV* cardiovascular, *HR* hazard ratio, *SASP* senescence-associated secretory phenotype, *OP* osteoporosis

RANKL inhibitors: Denosumab is a fully human monoclonal antibody that binds to RANK. By preventing the interaction between RANKL and its receptor RANK on osteoclast precursors and mature osteoclasts, denosumab profoundly suppresses osteoclast differentiation, activation, and survival.^206^ Administered via subcutaneous injection, it is convenient and has been shown to continuously increase BMD across multiple skeletal sites while significantly reducing fracture risk. However, abrupt discontinuation may result in a rebound increase in bone turnover markers and rapid bone loss^207^—a phenomenon now well-quantified. Following denosumab withdrawal, approximately 10% of patients experience multiple vertebral fractures, particularly those with a prior vertebral fracture history.^208^ Although rare (<0.5%), rebound hypercalcemia can also occur, typically between three and eleven months after the last dose. These complications are generally reversible upon re-treatment with either denosumab or bisphosphonates.^209^

Selective estrogen receptor modulators (SERMs): Raloxifene exerts estrogen-like effects on bone, inhibiting bone resorption and reducing vertebral fracture risk, without stimulating breast or endometrial tissues. These properties make it suitable for the prevention and treatment of postmenopausal osteoporosis.^210^ Calcitonin, due to insufficient evidence supporting long-term efficacy and concerns regarding potential cancer risk, now plays a secondary role and is primarily used for short-term relief of acute bone pain.^211^

Hormone replacement therapy (HRT): Estrogen-based hormone replacement therapy is a highly effective anti-resorptive agent for younger postmenopausal women (age ≤60 years) with high fracture risk, and low baseline risk for adverse malignant and thromboembolic events. HRT acts primarily by binding to estrogen receptors on osteoclasts, inducing their apoptosis and thereby suppressing bone resorption. This mechanism leads to sustained improvements in bone mineral density across all skeletal sites.^212^ Large-scale randomized controlled trials and their long-term follow-up studies have demonstrated that HRT significantly reduces the risk of vertebral, hip, and non-vertebral fractures.^213^ However, HRT is not recommended as a first-line therapy for older postmenopausal women without menopausal symptoms due to the increased absolute risks of stroke, venous thromboembolism, and, with long-term combined therapy, breast cancer. When prescribed, the lowest effective dose for the shortest duration necessary to control symptoms is recommended, with annual re-evaluation of risks and benefits.

#### Anabolic agents

PTH/PTHrP analogs: Teriparatide, a recombinant human PTH, preferentially stimulates bone formation when administered intermittently at low doses via subcutaneous injection. This anabolic effect is mediated primarily through activation of the PKA signaling pathway in osteoblasts. Teriparatide significantly improves BMD and bone microstructure and reduces the risk of both vertebral and non-vertebral fractures.^214^ The duration of teriparatide therapy is typically limited to 24 months, a precaution originally based on an increased incidence of osteosarcoma observed in high-dose, near-lifetime rat studies.^215^ However, long-term clinical trials and post-marketing surveillance extending up to 15 years have not demonstrated any increased risk of osteosarcoma in humans treated with teriparatide.^216^ Large observational studies using claims data linked to cancer registries have shown no increase in osteosarcoma incidence compared with unexposed populations or expected background rates, leading to the removal of the boxed warning from regulatory labels.^217^ Another PTHrP analog is Abaloparatide. Compared with teriparatide, it shows superior anti-fracture efficacy in major osteoporotic fractures (upper arm, wrist, hip, or clinical vertebrae) and has a lower risk of hypercalcemia.^218^

Sclerostin monoclonal antibody: Romosozumab inhibits sclerostin, a negative regulator of bone formation. By releasing inhibition of the Wnt/β-catenin signaling pathway, romosozumab robustly stimulates bone formation while simultaneously exerting moderate anti-resorptive effects.^219^ Treatment can substantially increase BMD and reduce fracture risk within a relatively short period (typically 12 months). Initial concerns about a potential increase in major adverse cardiovascular events (MACE), raised by a pivotal phase 3 trial, have been reassessed in subsequent real-world studies. A large cohort study using Japan’s national claims database (251 219 romosozumab users vs. 500 445 teriparatide users) found no statistically significant difference in MACE risk between romosozumab and teriparatide (adjusted HR 1.00; 95% CI 0.94–1.06), regardless of prior MACE history.^220^ Meta-analyses of randomized controlled trials have similarly shown no significant differences in cardiovascular events between romosozumab and placebo.^221^ Nevertheless, given the persistent clinical uncertainty, careful assessment of cardiovascular risk remains prudent before use, particularly in patients with a recent history of myocardial infarction or stroke.

#### Sequential treatment strategies

The availability of both anti-resorptive and anabolic agents has expanded therapeutic options for osteoporosis but has also introduced complexity regarding the optimal sequence of treatment. Given that anabolic agents achieve their maximal fracture-reduction benefits within 12–24 months and that their effects are not sustained after discontinuation, while anti-resorptive agents provide durable fracture protection with extended use, sequential therapy has emerged as the standard of care for patients at very high fracture risk.

From anabolic to anti-resorptive therapy (the anabolic-first strategy): Contemporary evidence strongly supports initiating treatment with an anabolic agent followed by an anti-resorptive agent in patients with very high fracture risk—defined as those with recent or multiple fragility fractures, very low BMD (T-score < −3.0), or fractures despite ongoing anti-resorptive therapy.^222^ This sequential approach capitalizes on the rapid and robust bone formation induced by anabolic agents and subsequently maintains the accrued bone mass with anti-resorptive therapy. A landmark meta-analysis of six randomized controlled trials enrolling 17 872 participants demonstrated that, compared with anti-resorptive monotherapy or placebo, initial anabolic therapy followed by anti-resorptive agents reduced vertebral fracture risk by 57%, clinical fracture risk by 38%, and hip fracture risk by 35%.^223^

From anti-resorptive to anabolic therapy: In patients who have been on long-term bisphosphonate therapy and remain at very high fracture risk, switching to an anabolic agent is recommended. However, bone turnover may be significantly suppressed after prolonged bisphosphonate use, potentially attenuating the initial response to anabolic therapy. In such cases, a washout period of several months before initiating an anabolic agent may be considered.^222^

Transition from denosumab: Denosumab discontinuation presents a unique clinical challenge due to rapid bone loss and increased vertebral fracture risk. Therefore, denosumab should never be discontinued without transition to an alternative anti-resorptive agent. When denosumab is stopped, a bisphosphonate should be administered promptly, typically within six months of the last denosumab injection, to mitigate rebound bone loss. For patients transitioning from denosumab to an anabolic agent, the optimal timing and sequence remain uncertain; current clinical practice often initiates the anabolic agent while continuing denosumab or shortly after the last denosumab dose to avoid an unprotected interval.^224^

#### Senolytic therapies

Senolytic agents: Senolytics represent an emerging therapeutic strategy targeting a core mechanism of bone aging: the accumulation of senescent cells.^225^ Senescent cells disrupt bone homeostasis through the secretion of SASP factors. Senolytic agents selectively induce apoptosis in senescent cells by targeting their anti-apoptotic pathways.^226^ Among the earliest studied combinations is dasatinib and quercetin (D + Q), which has been shown in aged mice and radiation-induced bone injury models to eliminate senescent osteoblasts, osteocytes, and other cell types, thereby restoring skeletal structure and function.^58,60,227^ The first phase 2 RCT of intermittent senolytic therapy (D + Q) in postmenopausal women (*n* = 60) was published in *Nature Medicine* (2024).^228^ The primary endpoint—change in the bone resorption marker CTX at 20 weeks—did not differ between groups (D + Q -4.1% vs. control -7.7%; *P* = 0.611). However, the bone formation marker P1NP increased significantly in the D + Q group at both 2 weeks (+16%, *P* = 0.020) and 4 weeks (+16%, *P* = 0.024). In exploratory analyses, women with high senescent cell burden (top tertile of T cell p16INK4a mRNA) showed concomitant increases in P1NP (+34%, *P* = 0.035) and reductions in CTX (−11%, *P* = 0.049) at 2 weeks, and significant radius BMD gains (+2.7%, *P* = 0.004) at 20 weeks.

SASP Modulators (Senomorphics): In addition to direct senescent clearance, senomorphics exert therapeutic effects by suppressing the secretion of SASP factors. For instance, JAK inhibitors such as ruxolitinib can mitigate age-related osteoporosis by inhibiting cytokines such as IL-6.^58^ Rapamycin and its analogs have also been reported to suppress SASP activity.^229^ Additionally, the natural flavonoid fisetin exhibits senolytic activity and has demonstrated potential benefits in improving osteoarthritis.^230^

### Emerging therapies

Beyond established pharmacotherapies, several innovative strategies are emerging to target the core mechanisms of bone aging. These approaches—stem cell therapy, exosome therapy, gene therapy, and smart biomaterials—differ substantially in their mechanism of action, stage of development, efficacy profile, and safety considerations. Rather than describing each in isolation, this section synthesizes the current evidence across strategies to guide future research priorities.

In terms of efficacy comparison, although direct head-to-head comparisons are lacking, preclinical studies using similar animal models allow for approximate effect size estimation. Gene therapy, particularly Wnt-pathway modulating gene silencers delivered via bone-targeted AAV vectors, has demonstrated the most robust bone regeneration, achieving >50% defect healing and systemic reversal of bone loss in osteoporotic mice following a single administration.^231^ Stem cell therapy has shown consistent but moderate efficacy, with allogeneic BMSC transplantation achieving approximately 40%–50% defect healing and improved BMD in osteoporotic models.^232,233^ Exosome therapy, while generally less potent than cell-based or gene-based approaches, offers distinct advantages in safety and scalability. For instance, MSC-derived exosomes carrying miR-29 reduced fracture healing time by approximately 30% in animal studies without the tumorigenic risk associated with stem cells.^40^

On the path to clinical translation, among these strategies, stem cell therapy has advanced farthest into clinical translation, with allogeneic BMSCs successfully used for segmental bone defect repair in humans, and adipose-derived stem cells entering early-phase trials for osteoporotic fractures.^234^ Gene therapy has achieved regulatory approval for osteoarthritis,^235^ and bone-targeted AAV-based approaches are now in late-stage preclinical development.^236^ Exosome therapy and smart biomaterials remain predominantly at the preclinical stage, though both have demonstrated promising safety profiles in small and large animal models.^237,238^

Regarding the trade-off between safety and effectiveness, a consistent pattern emerges across strategies: interventions with greater efficacy often carry higher safety risks. Gene therapy offers durable, single-administration efficacy but raises concerns about insertional mutagenesis, immunogenicity, and off-target gene editing. Stem cell therapy faces challenges of post-transplantation cell survival, batch-to-batch variability, and potential tumorigenicity. Exosome therapy exhibits the most favorable safety profile (low immunogenicity, no live cells, no viral vectors) but requires optimization of targeting efficiency and half-life extension in circulation.^239^ Smart biomaterials provide structural support and controlled local delivery but lack the biological complexity to fully recapitulate native bone tissue.^240^

## Challenges and future directions

### Ongoing challenges

#### Depth and integration of mechanistic elucidation

Current research on bone aging largely focuses on changes within individual cell types or isolated signaling pathways. However, a defining feature of bone aging is the systemic failure of communication networks. Several fundamental questions remain inadequately addressed: How does the functional decline of osteocytes, as mechanosensors and endocrine regulators, precisely affect osteoblast-osteoclast coupling? What is the causal relationship between impaired osteogenic-adipogenic lineage commitment of BMSCs and the accumulation of marrow adipose tissue? How is crosstalk between bone and other organs, including muscle, brain, gut, and the immune system, altered during aging? Existing studies often offer a fragmented view, lacking a unified framework that temporally and spatially integrates molecular, cellular, tissue, and systemic levels.

#### The gap between research models and human bone aging

Bone aging research relies predominantly on animal models, particularly mice. However, substantial differences exist between murine and human skeletal physiology and aging trajectories. Humans exhibit extensive Haversian remodeling, a prolonged period of skeletal maturation, and distinct patterns of age-related bone loss. Conventional aged mouse models fail to replicate the decades-long process of bone accrual and gradual degeneration observed in humans. Furthermore, in vitro cell culture systems cannot adequately reproduce the complex three-dimensional microenvironment, mechanical loading, and systemic hormonal regulation present in vivo. These limitations contribute to the frequent failure of therapeutics that show efficacy in preclinical models to translate successfully into human clinical trials.

#### Scarcity and heterogeneity of bone aging biomarkers

The premise of precision medicine is accurate assessment. Yet, clinical evaluation of bone aging relies heavily on BMD, a delayed and incomplete indicator. BMD does not capture critical factors determining bone fragility, such as bone turnover rate, microarchitectural deterioration, and declines in mechanical properties. There is an urgent need to identify multimodal biomarker panels capable of capturing the biological process of bone aging in an early, dynamic, and comprehensive manner. Moreover, the molecular basis underlying the significant individual heterogeneity and sexual dimorphism in bone aging, and how to achieve risk stratification and personalized intervention, remains poorly understood.

#### Bottlenecks in intervention strategies

Current first-line anti-osteoporotic therapies primarily work by potently suppressing bone resorption, raising concerns about excessive inhibition of bone remodeling during long-term use. Although anabolic agents can increase bone mass, their application is limited by restricted treatment duration and high cost. More fundamentally, most existing therapies are symptomatic rather than causal, failing to address the underlying drivers of bone aging. Emerging interventions based on the biology of aging, such as interventions targeting cellular senescence, mitochondrial dysfunction, defective autophagy, and stem cell exhaustion, offer considerable promise. However, they face significant challenges, including targeted delivery efficiency, tissue specificity, and long-term safety.

#### Sociomedical issues

Skeletal aging represents a classic “silent epidemic”: bone loss progresses asymptomatically until a fragility fracture occurs, often with devastating consequences. This delayed clinical manifestation contributes to insufficient public awareness, delayed policy responses, and chronic underinvestment in preventive strategies. Although fragility fractures impose enormous direct and indirect economic burdens through medical costs, rehabilitation, and loss of productivity, these downstream consequences have not been effectively translated into incentives for upstream prevention. Furthermore, the rate and impact of bone aging are profoundly influenced by social determinants of health, including socioeconomic status, gender and age discrimination, and geographic disparities, resulting in significant health inequities.

### Future directions

#### Establishing multi-omics integrated research paradigms

Future research must transcend the perspective of isolated pathways and shift towards constructing a comprehensive bone aging atlas. This requires integrating genomics, epigenomics, proteomics, metabolomics, and single-cell sequencing technologies to systematically characterize dynamic interaction networks among different cell types within bone tissue during aging. A key focus will be elucidating how bone, as an endocrine organ, communicates with and is influenced by distant organs over time. Identifying central regulatory hubs and critical network nodes will provide a conceptual foundation for the development of multi-target, mechanism-based interventions.

#### Developing organoid and computational models

To address the limitations of traditional animal models, future efforts should prioritize the development of advanced in vitro systems and digital models that more closely mimic human physiology. Human induced pluripotent stem cells can be used to generate three-dimensional organoids or “bone-on-a-chip” platforms incorporating bone, bone marrow, vasculature, and immune components, thereby simulating the complex microenvironment and aging process of human bone. In parallel, integrating clinical imaging data, biomechanical measurements, and multi-omics datasets will facilitate the development of digital twins and artificial intelligence-driven predictive models of bone aging. These approaches have the potential to significantly enhance the efficiency and precision of translating basic discoveries into clinical applications and reduce the failure rate in drug development.

#### Establishing multimodal, dynamic precision risk assessment systems

Moving beyond the reliance on BMD alone, future efforts should establish precision assessment systems that integrate structural, functional, and molecular information. This includes the use of advanced imaging techniques such as high-resolution peripheral quantitative computed tomography to quantitatively assess bone microstructure; developing novel biomarker panels based on blood or urine that can dynamically reflect bone turnover, cellular senescence status, and metabolic signatures; and incorporating genetic risk scores and clinical risk factors. Ultimately, artificial intelligence algorithms will be employed to integrate multidimensional data, enabling early warning, precise stratification, and dynamic monitoring of the bone aging rate, fracture risk, and therapeutic response.

#### Developing therapies targeting core mechanisms of bone aging

Future therapeutic strategies should directly target the fundamental biological mechanisms driving skeletal aging. These include the development of bone-targeted senolytic drugs to selectively eliminate senescent cells and ameliorate the pro-inflammatory bone microenvironment. At the stem cell level, strategies include restoring the regenerative potential of aged stem cells through epigenetic reprogramming, mitochondrial function enhancement, or targeted drug delivery. Systemic metabolic and immune modulation will focus on interventions against age-related chronic inflammation and metabolic disorders to systemically improve skeletal health. Concurrently, the development of smart delivery systems, including nanocarriers, is essential to ensure these novel therapies act precisely and safely on bone tissue.

#### Promoting equity-centered public health strategies focused on prevention

Future public health initiatives should prioritize the implementation of comprehensive lifespan-oriented bone health action plans. These should incorporate bone density screening and risk assessment into routine health check-ups. Additionally, digital health and wearable should be leveraged for community- and household-based remote monitoring and personalized exercise and nutritional guidance. Crucially, health equity must be placed at the core. Through targeted screening, health education, and the allocation of medical resources, special attention must be provided to reduce skeletal health disparities caused by socioeconomic differences, ensuring that intervention measures benefit all populations.

## Summary

Bone aging represents a progressive disruption of skeletal homeostasis at macroscopic, microscopic, and systemic levels. Macroscopically, it manifests as bone loss, deterioration of bone microstructure, and a decline in biomechanical properties. Microscopically, it involves genetic alterations, epigenetic dysregulation, oxidative stress, dysfunction of key signaling pathways, and the emergence of the senescence-associated secretory phenotype. At the systemic level, it is closely linked to chronic inflammation, hormonal changes, and alterations in the extracellular matrix.

Current intervention strategies encompass lifestyle modifications, anti-resorptive and anabolic agents, and emerging strategies such as senolytic agents and regenerative medicine approaches. However, challenges remain, including insufficient integration of underlying mechanisms, limitations of existing models, scarcity of sensitive and specific biomarkers, restricted therapeutic precision, and persistent sociomedical barriers.

Future research necessitates the development of multi-omics integrated aging atlases, organoid and computational models, multimodal risk assessment systems, precision therapies targeting core mechanisms, and the promotion of prevention-centered public health strategies. These efforts are crucial for providing the scientific foundation and clinical translation pathways to achieve healthy skeletal aging.

## References

[1] Johnell, O. & Kanis, J. A. An estimate of the worldwide prevalence and disability associated with osteoporotic fractures. *Osteoporos. Int.* **17**, 1726–1733 (2006). 16983459 10.1007/s00198-006-0172-4

[2] Welc, S. S., Brotto, M., White, K. E. & Bonewald, L. F. Aging: a struggle for beneficial to overcome negative factors made by muscle and bone. *Mech. Ageing Dev.* **224**, 112039 (2025). 39952614 10.1016/j.mad.2025.112039 PMC11893237

[3] Barkaoui, A., Ben Kahla, R., Merzouki, T. & Hambli, R. Age and gender effects on bone mass density variation: finite elements simulation. *Biomech. Model Mechanobiol.* **16**, 521–535 (2017). 27659482 10.1007/s10237-016-0834-x

[4] Riggs, B. L. et al. Differential changes in bone mineral density of the appendicular and axial skeleton with aging: relationship to spinal osteoporosis. *J. Clin. Investig.* **67**, 328–335 (1981). 7462421 10.1172/JCI110039 PMC370572

[5] Cui, J., Shibata, Y., Zhu, T., Zhou, J. & Zhang, J. Osteocytes in bone aging: advances, challenges, and future perspectives. *Ageing Res. Rev.* **77**, 101608 (2022). 35283289 10.1016/j.arr.2022.101608

[6] Augat, P. & Schorlemmer, S. The role of cortical bone and its microstructure in bone strength. *Age Ageing* **35**, ii27–ii31 (2006). 16926200 10.1093/ageing/afl081

[7] Seeman, E. & Delmas, P. D. Bone quality-the material and structural basis of bone strength and fragility. *N. Engl. J. Med.* **354**, 2250–2261 (2006). 16723616 10.1056/NEJMra053077

[8] Chartier, S. R., Mitchell, S. A. T., Majuta, L. A. & Mantyh, P. W. The changing sensory and sympathetic innervation of the young, adult and aging mouse femur. *Neuroscience* **387**, 178–190 (2018). 29432884 10.1016/j.neuroscience.2018.01.047 PMC6086773

[9] Feingold, M., Nelson, D. A. & Parfitt, A. M. Composite index of skeletal mass: principal components analysis of regional bone mineral densities. *J. Bone Miner. Res.* **7**, 89–96 (1992). 1549962 10.1002/jbmr.5650070113

[10] Seeman, E. Pathogenesis of bone fragility in women and men. *Lancet* **359**, 1841–1850 (2002). 12044392 10.1016/S0140-6736(02)08706-8

[11] Seeman, E. Age- and menopause-related bone loss compromise cortical and trabecular microstructure. *J. Gerontol. A Biol. Sci. Med Sci.* **68**, 1218–1225 (2013). 23833200 10.1093/gerona/glt071

[12] Mosekilde, L., Mosekilde, L. & Danielsen, C. C. Biomechanical competence of vertebral trabecular bone in relation to ash density and age in normal individuals. *Bone* **8**, 79–85 (1987). 3593611 10.1016/8756-3282(87)90074-3

[13] Martin, B. Aging and strength of bone as a structural material. *Calcif. Tissue Int.* **53**, S34–S40 (1993). 8275378 10.1007/BF01673400

[14] Boskey, A. L. & Coleman, R. Aging and bone. *J. Dent. Res.* **89**, 1333–1348 (2010). 20924069 10.1177/0022034510377791 PMC2991386

[15] Ballhause, T. M. et al. Spatio-temporal characterization of compositional and cellular properties in the murine fracture callus. *Acta Biomater.* **206**, 150–159 (2025). 10.1016/j.actbio.2025.09.049 41038549

[16] Yu, Y. E., Hu, Y. J., Zhou, B., Wang, J. & Guo, X. E. Microstructure determines apparent-level mechanics despite tissue-level anisotropy and heterogeneity of individual plates and rods in normal human trabecular bone. *J. Bone Miner. Res.* **36**, 1796–1807 (2021). 33989436 10.1002/jbmr.4338

[17] Koehne, T. et al. Trends in trabecular architecture and bone mineral density distribution in 152 individuals aged 30-90 years. *Bone* **66**, 31–38 (2014). 24859568 10.1016/j.bone.2014.05.010

[18] Saito, M. & Marumo, K. Collagen Cross-links as a determinant of bone quality. In *Osteoporosis in Orthopedics: Assessment and Therapeutic Options* (eds Shimada, Y. & Miyakoshi, N.) 35–54. 10.1007/978-4-431-55778-4_3 (Springer Japan, 2016)

[19] Saito, M. & Marumo, K. Collagen cross-links as a determinant of bone quality: a possible explanation for bone fragility in aging, osteoporosis, and diabetes mellitus. *Osteoporos. Int.* **21**, 195–214 (2010). 19760059 10.1007/s00198-009-1066-z

[20] Casella, G. et al. Transcriptome signature of cellular senescence. *Nucleic Acids Res.* **47**, 7294–7305 (2019). 31251810 10.1093/nar/gkz555 PMC6698740

[21] Purcell, M., Kruger, A. & Tainsky, M. A. Gene expression profiling of replicative and induced senescence. *Cell Cycle* **13**, 3927–3937 (2014). 25483067 10.4161/15384101.2014.973327 PMC4615143

[22] Hernandez-Segura, A. et al. Unmasking transcriptional heterogeneity in senescent cells. *Curr. Biol.* **27**, 2652–2660.e4 (2017). 28844647 10.1016/j.cub.2017.07.033 PMC5788810

[23] Kim, Y.-M. et al. Implications of time-series gene expression profiles of replicative senescence. *Aging Cell* **12**, 622–634 (2013). 23590226 10.1111/acel.12087

[24] Jochems, F. et al. The Cancer SENESCopedia: a delineation of cancer cell senescence. *Cell Rep.* **36**, 109441 (2021). 34320349 10.1016/j.celrep.2021.109441 PMC8333195

[25] de Boer, J. et al. Premature aging in mice deficient in DNA repair and transcription. *Science* **296**, 1276–1279 (2002). 11950998 10.1126/science.1070174

[26] Rasheed, N., Wang, X., Niu, Q.-T., Yeh, J. & Li, B. Atm-deficient mice: an osteoporosis model with defective osteoblast differentiation and increased osteoclastogenesis. *Hum. Mol. Genet.* **15**, 1938–1948 (2006). 16644862 10.1093/hmg/ddl116

[27] Ruzankina, Y. et al. Deletion of the developmentally essential gene ATR in adult mice leads to age-related phenotypes and stem cell loss. *Cell Stem Cell* **1**, 113–126 (2007). 18371340 10.1016/j.stem.2007.03.002 PMC2920603

[28] Chen, Q. et al. DNA damage drives accelerated bone aging via an NF-κB-dependent mechanism. *J. Bone Miner. Res.* **28**, 1214–1228 (2013). 23281008 10.1002/jbmr.1851 PMC3662975

[29] Chandra, A. et al. Proteasome inhibitor bortezomib is a novel therapeutic agent for focal radiation-induced osteoporosis. *FASEB J.* **32**, 52–62 (2018). 28860152 10.1096/fj.201700375R PMC5731129

[30] Rivadeneira, F. et al. Twenty bone-mineral-density loci identified by large-scale meta-analysis of genome-wide association studies. *Nat. Genet.* **41**, 1199–1206 (2009). 19801982 10.1038/ng.446 PMC2783489

[31] Wehmeyer, C. et al. Sclerostin inhibition promotes TNF-dependent inflammatory joint destruction. *Sci. Transl. Med.* **8**, 330ra35 (2016). 27089204 10.1126/scitranslmed.aac4351

[32] Sims, A.-M. et al. Genetic analyses in a sample of individuals with high or low BMD shows association with multiple Wnt pathway genes. *J. Bone Miner. Res.* **23**, 499–506 (2008). 18021006 10.1359/jbmr.071113

[33] Saul, D. et al. A new gene set identifies senescent cells and predicts senescence-associated pathways across tissues. *Nat. Commun.* **13**, 4827 (2022). 35974106 10.1038/s41467-022-32552-1 PMC9381717

[34] Chandra, A. et al. Bone marrow adiposity in models of radiation- and aging-related bone loss is dependent on cellular senescence. *J. Bone Miner. Res.* **37**, 997–1011 (2022). 35247283 10.1002/jbmr.4537 PMC9526878

[35] Fuggle, N. R., Laskou, F., Harvey, N. C. & Dennison, E. M. A review of epigenetics and its association with ageing of muscle and bone. *Maturitas* **165**, 12–17 (2022). 35841774 10.1016/j.maturitas.2022.06.014

[36] Soerensen, M., Figeac, F., Christensen, K. & Kassem, M. Epigenetic clocks as biomarkers for bone aging: evidence from a twin study. *Aging Cell* **24**, e70204 (2025). 40947307 10.1111/acel.70204 PMC12507416

[37] Alshammari, R. A. et al. Integrative epigenomic and targeted protein analysis in MRONJ: correlating DNA methylation with bone biomarkers. *Int. J. Mol. Sci.* **26**, 11208 (2025). 41303691 10.3390/ijms262211208 PMC12653879

[38] Reppe, S. et al. Distinct DNA methylation profiles in bone and blood of osteoporotic and healthy postmenopausal women. *Epigenetics* **12**, 674–687 (2017). 28650214 10.1080/15592294.2017.1345832 PMC5687328

[39] Voisin, S. et al. An epigenetic clock for human skeletal muscle. *J. Cachexia Sarcopenia Muscle* **11**, 887–898 (2020). 32067420 10.1002/jcsm.12556 PMC7432573

[40] Lin, Q. et al. Delivery of miR-29a/29c-3p by serum exosomes promotes osteogenesis through TET3-dependent Sox9 demethylation and PI3K/Akt activation. *J. Nanobiotechnol.* **23**, 751 (2025). 10.1186/s12951-025-03743-x PMC12670819 41327200

[41] Cheng, K. et al. MicroRNA-874-3p is a potential contributor to primary hyperparathyroidism-induced osteoporosis. *Clin. Interv. Aging* **20**, 2079–2089 (2025). 41282376 10.2147/CIA.S538129 PMC12640103

[42] Huang, B. et al. KDM3A and KDM4C regulate mesenchymal stromal cell senescence and bone aging via condensin-mediated heterochromatin reorganization. *iScience* **21**, 375–390 (2019). 31704649 10.1016/j.isci.2019.10.041 PMC6888768

[43] Wu, L. et al. Integrative analysis reveals synergistic regulation of Sp7 by BRD9 and Wnt/β-catenin signaling during osteogenic differentiation. *Commun. Biol.* **9**, 23 (2025). 41326797 10.1038/s42003-025-09278-z PMC12775129

[44] Liu, F. et al. S-sulfhydration of SIRT3 combats BMSC senescence and ameliorates osteoporosis via stabilizing heterochromatic and mitochondrial homeostasis. *Pharm. Res.* **192**, 106788 (2023). 10.1016/j.phrs.2023.106788 37146925

[45] Li, M. et al. Genistein mitigates senescence of bone marrow mesenchymal stem cells via ERRα-mediated mitochondrial biogenesis and mitophagy in ovariectomized rats. *Redox Biol.* **61**, 102649 (2023). 36871183 10.1016/j.redox.2023.102649 PMC9995482

[46] Sun, Y. et al. Macrophage-osteoclast associations: origin, polarization, and subgroups. *Front. Immunol.* **12**, 778078 (2021). 34925351 10.3389/fimmu.2021.778078 PMC8672114

[47] Gao, J. et al. SIRT3/SOD2 maintains osteoblast differentiation and bone formation by regulating mitochondrial stress. *Cell Death Differ.* **25**, 229–240 (2018). 28914882 10.1038/cdd.2017.144 PMC5762839

[48] Zhu, C. et al. Autophagy in bone remodeling: a regulator of oxidative stress. *Front. Endocrinol.* **13**, 898634 (2022). 10.3389/fendo.2022.898634 PMC9279723 35846332

[49] Zahan, O.-M., Serban, O., Gherman, C. & Fodor, D. The evaluation of oxidative stress in osteoarthritis. *Med. Pharm. Rep.* **93**, 12–22 (2020). 32133442 10.15386/mpr-1422 PMC7051818

[50] Li, J. et al. Pyrroloquinoline quinone alleviates natural aging-related osteoporosis via a novel MCM3-Keap1-Nrf2 axis-mediated stress response and Fbn1 upregulation. *Aging Cell* **22**, e13912 (2023). 37365714 10.1111/acel.13912 PMC10497824

[51] Yuan, H. et al. Ganoderic acid D prevents oxidative stress-induced senescence by targeting 14-3-3ε to activate CaM/CaMKII/NRF2 signaling pathway in mesenchymal stem cells. *Aging Cell* **21**, e13686 (2022). 35929187 10.1111/acel.13686 PMC9470892

[52] Sun, K. et al. The role of the sirtuin family in cartilage and osteoarthritis: molecular mechanisms and therapeutic targets. *Arthritis Res. Ther.* **24**, 286 (2022). 36585687 10.1186/s13075-022-02983-8 PMC9805245

[53] Zhou, Z. et al. Protective effect of SIRT1 activator on the knee with osteoarthritis. *Front. Physiol.* **12**, 661852 (2021). 33927645 10.3389/fphys.2021.661852 PMC8076744

[54] Zhang, J., Yu, H., Man, M.-Q. & Hu, L. Aging in the dermis: fibroblast senescence and its significance. *Aging Cell* **23**, e14054 (2024). 38040661 10.1111/acel.14054 PMC10861215

[55] Jha, S. K. et al. Cellular senescence in lung cancer: molecular mechanisms and therapeutic interventions. *Ageing Res. Rev.* **97**, 102315 (2024). 38679394 10.1016/j.arr.2024.102315

[56] Fang, C.-L., Liu, B. & Wan, M. Bone-SASP’ in skeletal aging. *Calcif. Tissue Int.* **113**, 68–82 (2023). 37256358 10.1007/s00223-023-01100-4 PMC10230496

[57] Farr, J. N. et al. Identification of senescent cells in the bone microenvironment. *J. Bone Miner. Res.* **31**, 1920–1929 (2016). 27341653 10.1002/jbmr.2892 PMC5289710

[58] Chandra, A. et al. Targeted reduction of senescent cell burden alleviates focal radiotherapy-related bone loss. *J. Bone Miner. Res.* **35**, 1119–1131 (2020). 32023351 10.1002/jbmr.3978 PMC7357625

[59] Hennrich, M. L. et al. Cell-specific proteome analyses of human bone marrow reveal molecular features of age-dependent functional decline. *Nat. Commun.* **9**, 4004 (2018). 30275468 10.1038/s41467-018-06353-4 PMC6167374

[60] Farr, J. N. et al. Targeting cellular senescence prevents age-related bone loss in mice. *Nat. Med.* **23**, 1072–1079 (2017). 28825716 10.1038/nm.4385 PMC5657592

[61] Lee, J. et al. Cabozantinib, an anti-aging agent, prevents bone loss in estrogen-deficient mice by suppressing senescence-associated secretory phenotype factors. *Int. J. Mol. Sci.* **26**, 7123 (2025). 40806256 10.3390/ijms26157123 PMC12346361

[62] Farr, J. N. et al. Effects of age and estrogen on skeletal gene expression in humans as assessed by RNA sequencing. *PLoS One* **10**, e0138347 (2015). 26402159 10.1371/journal.pone.0138347 PMC4581624

[63] Movérare-Skrtic, S. et al. Osteoblast-derived WNT16 represses osteoclastogenesis and prevents cortical bone fragility fractures. *Nat. Med.* **20**, 1279–1288 (2014). 25306233 10.1038/nm.3654 PMC4392888

[64] Yu, B. et al. Wnt4 signaling prevents skeletal aging and inflammation by inhibiting nuclear factor-κB. *Nat. Med.* **20**, 1009–1017 (2014). 25108526 10.1038/nm.3586 PMC4159424

[65] Cosman, F. et al. Romosozumab treatment in postmenopausal women with osteoporosis. *N. Engl. J. Med.* **375**, 1532–1543 (2016). 27641143 10.1056/NEJMoa1607948

[66] Bai, J. et al. Gαi1/3 regulates Wnt/β-Catenin signaling to promote osteogenesis and bone formation. *J. Bone Miner. Res.* **41**, 46–59 (2025). 41273070 10.1093/jbmr/zjaf143

[67] Song, J. et al. FOXO-regulated OSER1 reduces oxidative stress and extends lifespan in multiple species. *Nat. Commun.* **15**, 7144 (2024). 39164296 10.1038/s41467-024-51542-z PMC11336091

[68] Wang, G. et al. Roburic acid attenuates osteoclastogenesis and bone resorption by targeting RANKL-induced intracellular signaling pathways. *J. Cell Physiol.* **237**, 1790–1803 (2022). 34796915 10.1002/jcp.30642

[69] Lorthongpanich, C. et al. YAP as a key regulator of adipo-osteogenic differentiation in human MSCs. *Stem Cell Res. Ther.* **10**, 402 (2019). 31852542 10.1186/s13287-019-1494-4 PMC6921580

[70] Sladitschek-Martens, H. L. et al. YAP/TAZ activity in stromal cells prevents ageing by controlling cGAS-STING. *Nature* **607**, 790–798 (2022). 35768505 10.1038/s41586-022-04924-6 PMC7613988

[71] Li, Z., Lin, J., Wu, J., Suo, J. & Wang, Z. The Hippo signalling pathway in bone homeostasis: under the regulation of mechanics and aging. *Cell Prolif.* **57**, e13652 (2024). 38700015 10.1111/cpr.13652 PMC11471399

[72] Coffin, J. D., Homer-Bouthiette, C. & Hurley, M. M. Fibroblast growth factor 2 and its receptors in bone biology and disease. *J. Endocr. Soc.* **2**, 657–671 (2018). 29942929 10.1210/js.2018-00105 PMC6009610

[73] Kir, S. et al. FGF19 as a postprandial, insulin-independent activator of hepatic protein and glycogen synthesis. *Science* **331**, 1621–1624 (2011). 21436455 10.1126/science.1198363 PMC3076083

[74] Zhao, Y.-X. et al. Association between bile acid metabolism and bone mineral density in postmenopausal women. *Clinics* **75**, e1486 (2020). 32187280 10.6061/clinics/2020/e1486 PMC7061317

[75] Bär, L. et al. Insulin suppresses the production of fibroblast growth factor 23 (FGF23). *Proc. Natl. Acad. Sci. USA* **115**, 5804–5809 (2018). 29760049 10.1073/pnas.1800160115 PMC5984514

[76] Rupp, T. et al. High FGF23 levels are associated with impaired trabecular bone microarchitecture in patients with osteoporosis. *Osteoporos. Int.* **30**, 1655–1662 (2019). 31044263 10.1007/s00198-019-04996-7

[77] Wu, Y.-T. et al. Lower serum fibroblast growth factor 21 levels are associated with normal lumbar spine bone mineral density in hemodialysis patients. *Int. J. Environ. Res. Public Health* **17**, 1938 (2020). 32188054 10.3390/ijerph17061938 PMC7143095

[78] Hu, W. et al. Fibroblast growth factor 21 is associated with bone mineral density, but not with bone turnover markers and fractures in chinese postmenopausal women. *J. Clin. Densitom.* **22**, 179–184 (2019). 30228048 10.1016/j.jocd.2018.08.005

[79] Valdes, A. M. et al. Telomere length in leukocytes correlates with bone mineral density and is shorter in women with osteoporosis. *Osteoporos. Int.* **18**, 1203–1210 (2007). 17347788 10.1007/s00198-007-0357-5

[80] Bekaert, S. et al. Telomere length versus hormonal and bone mineral status in healthy elderly men. *Mech. Ageing Dev.* **126**, 1115–1122 (2005). 15967485 10.1016/j.mad.2005.04.007

[81] Sanders, J. L. et al. Leukocyte telomere length is not associated with BMD, osteoporosis, or fracture in older adults: results from the health, aging and body composition study. *J. Bone Miner. Res.* **24**, 1531–1536 (2009). 19338455 10.1359/JBMR.090318 PMC2730927

[82] Tang, N. L. S. et al. The effect of telomere length, a marker of biological aging, on bone mineral density in elderly population. *Osteoporos. Int.* **21**, 89–97 (2010). 19436937 10.1007/s00198-009-0948-4

[83] Kirk, B. et al. Leukocyte telomere length is associated with MRI-thigh fat-free muscle volume: data from 16 356 UK Biobank adults. *J. Cachexia Sarcopenia Muscle* **15**, 1157–1166 (2024). 38553835 10.1002/jcsm.13461 PMC11154769

[84] Kalyan, S. et al. Premature spinal bone loss in women living with HIV is associated with shorter leukocyte telomere length. *Int. J. Environ. Res. Public Health* **15**, 1018 (2018). 29783641 10.3390/ijerph15051018 PMC5982057

[85] Kirk, B., Kuo, C.-L., Xiang, M. & Duque, G. Associations between leukocyte telomere length and osteosarcopenia in 20,400 adults aged 60 years and over: data from the UK Biobank. *Bone* **161**, 116425 (2022). 35489708 10.1016/j.bone.2022.116425 PMC11551922

[86] Pignolo, R. J. et al. Defects in telomere maintenance molecules impair osteoblast differentiation and promote osteoporosis. *Aging Cell* **7**, 23–31 (2008). 18028256 10.1111/j.1474-9726.2007.00350.x PMC2394673

[87] Guerrero-López, R. et al. Premature ageing of lung alveoli and bone marrow cells from Terc deficient mice with different telomere lengths. *Sci. Rep.* **15**, 6102 (2025). 39971959 10.1038/s41598-025-90246-2 PMC11840044

[88] von Zglinicki, T., Saretzki, G., Döcke, W. & Lotze, C. Mild hyperoxia shortens telomeres and inhibits proliferation of fibroblasts: a model for senescence? *Exp. Cell Res.* **220**, 186–193 (1995). 7664835 10.1006/excr.1995.1305

[89] Lavhale, M. P., Mandlik, S. K., Shinde, V. M. & Mandlik, D. S. Nrf2 signaling in bone health: unlocking new avenues for osteoporosis management. *Inflammopharmacology* **33**, 6419–6455 (2025). 41075071 10.1007/s10787-025-01985-7

[90] Deng, Y. et al. SIRT3-PINK1-PKM2 axis prevents osteoarthritis via mitochondrial renewal and metabolic switch. *Bone Res.* **13**, 36 (2025). 40087281 10.1038/s41413-025-00413-4 PMC11909255

[91] Zeng, X. et al. Matrix stiffness promotes DRP1-mediated myofibroblast senescence to drive silica-induced pulmonary fibrosis. *Aging Cell* **24**, e70275 (2025). 41104695 10.1111/acel.70275 PMC12686568

[92] Liu, J., Zhang, X., Hou, H., Ouyang, J. & Dai, J. Advances in osteoblast and mitochondrial dynamics and their transfer in osteoporosis. *J. Cell Mol. Med.* **28**, e70299 (2024). 39700051 10.1111/jcmm.70299 PMC11657594

[93] Cai, W. et al. STING regulates aging-related osteoporosis by mediating the Hk2-Vdac1 mitochondrial axis. *Free Radic. Biol. Med.* **225**, 1–14 (2024). 39326680 10.1016/j.freeradbiomed.2024.09.031

[94] Volodarsky, M. et al. A deletion mutation in TMEM38B associated with autosomal recessive osteogenesis imperfecta. *Hum. Mutat.* **34**, 582–586 (2013). 23316006 10.1002/humu.22274

[95] Cabral, W. A. et al. Absence of the ER cation channel TMEM38B/TRIC-B disrupts intracellular calcium homeostasis and dysregulates collagen synthesis in recessive osteogenesis imperfecta. *PLoS Genet.* **12**, e1006156 (2016). 27441836 10.1371/journal.pgen.1006156 PMC4956114

[96] Webb, E. A. et al. Phenotypic spectrum in osteogenesis imperfecta due to mutations in TMEM38B: unraveling a complex cellular defect. *J. Clin. Endocrinol. Metab.* **102**, 2019–2028 (2017). 28323974 10.1210/jc.2016-3766 PMC5470761

[97] Zhong, M., Wu, Z., Chen, Z., Ren, Q. & Zhou, J. Advances in the interaction between endoplasmic reticulum stress and osteoporosis. *Biomed. Pharmacother.* **165**, 115134 (2023). 37437374 10.1016/j.biopha.2023.115134

[98] Son, H.-E., Kim, E.-J. & Jang, W.-G. Curcumin induces osteoblast differentiation through mild-endoplasmic reticulum stress-mediated such as BMP2 on osteoblast cells. *Life Sci.* **193**, 34–39 (2018). 29223538 10.1016/j.lfs.2017.12.008

[99] Suzuki, R. et al. Intracellular accumulation of advanced glycation end products induces osteoblast apoptosis via endoplasmic reticulum stress. *J. Bone Miner. Res.* **35**, 1992–2003 (2020). 32427355 10.1002/jbmr.4053

[100] Zhu, T. & Yuan, L.-Q. Complex relationship between proton pump inhibitor use and bone health. *Am. J. Gastroenterol.* **112**, 651 (2017). 28381836 10.1038/ajg.2017.24

[101] Tao, Z.-S., Ma, T. & Yang, M. Cyclosporine a inhibits bone regeneration and induces bone loss in a rat model. *Int. Immunopharmacol.* **132**, 111951 (2024). 38552293 10.1016/j.intimp.2024.111951

[102] Chen, X. et al. Palmitic acid induces lipid droplet accumulation and senescence in nucleus pulposus cells via ER-stress pathway. *Commun. Biol.* **7**, 539 (2024). 38714886 10.1038/s42003-024-06248-9 PMC11076507

[103] Chalil, S. et al. Increased endoplasmic reticulum stress in mouse osteocytes with aging alters Cox-2 response to mechanical stimuli. *Calcif. Tissue Int.* **96**, 123–128 (2015). 25539857 10.1007/s00223-014-9944-6

[104] Zheng, B. et al. S1P regulates intervertebral disc aging by mediating endoplasmic reticulum-mitochondrial calcium ion homeostasis. *JCI Insight* **9**, e177789 (2024). 39316443 10.1172/jci.insight.177789 PMC11601718

[105] Yin, M., Zheng, X. & Shi, L. Targeting p38 MAPK: a potential bridge between ER stress and age-related bone loss. *Cell Signal* **127**, 111549 (2025). 39638139 10.1016/j.cellsig.2024.111549

[106] Zheng, J., Gao, Y., Lin, H., Yuan, C. & Keqianzhi Enhanced autophagy suppresses inflammation-mediated bone loss through ROCK1 signaling in bone marrow mesenchymal stem cells. *Cells Dev.* **167**, 203687 (2021). 34058434 10.1016/j.cdev.2021.203687

[107] Hayashi, C. et al. miR-1260b inhibits periodontal bone loss by targeting ATF6β-mediated regulation of ER stress. *Front. Cell Dev. Biol.* **10**, 1061216 (2022). 36531939 10.3389/fcell.2022.1061216 PMC9748617

[108] Zhang, Y. et al. CXCR4/CXCL12 axis counteracts hematopoietic stem cell exhaustion through selective protection against oxidative stress. *Sci. Rep.* **6**, 37827 (2016). 27886253 10.1038/srep37827 PMC5122894

[109] Wang, B., Dong, Y., Tian, Z., Chen, Y. & Dong, S. The role of dendritic cells derived osteoclasts in bone destruction diseases. *Genes Dis.* **8**, 401–411 (2021). 34179305 10.1016/j.gendis.2020.03.009 PMC8209356

[110] van der Kraan, A. G. J. et al. HSP90 inhibitors enhance differentiation and MITF (microphthalmia transcription factor) activity in osteoclast progenitors. *Biochem. J.* **451**, 235–244 (2013). 23379601 10.1042/BJ20121626

[111] Zhou, J., Fujiwara, T., Ye, S., Li, X. & Zhao, H. Ubiquitin E3 ligase LNX2 is critical for osteoclastogenesis in vitro by regulating M-CSF/RANKL signaling and Notch2. *Calcif. Tissue Int* **96**, 465–475 (2015). 25712254 10.1007/s00223-015-9967-7 PMC4730947

[112] Lee, K. et al. Blocking of the ubiquitin-proteasome system prevents inflammation-induced bone loss by accelerating M-CSF receptor c-Fms degradation in osteoclast differentiation. *Int. J. Mol. Sci.* **18**, 2054 (2017). 28946669 10.3390/ijms18102054 PMC5666736

[113] Li, M. et al. Gasdermin D maintains bone mass by rewiring the endo-lysosomal pathway of osteoclastic bone resorption. *Dev. Cell* **57**, 2365–2380.e8 (2022). 36243012 10.1016/j.devcel.2022.09.013

[114] Li, Y., Su, J., Sun, W., Cai, L. & Deng, Z. AMP-activated protein kinase stimulates osteoblast differentiation and mineralization through autophagy induction. *Int. J. Mol. Med.* **41**, 2535–2544 (2018). 29484369 10.3892/ijmm.2018.3498 PMC5846672

[115] Wang, J. et al. The role of autophagy in bone metabolism and clinical significance. *Autophagy* **19**, 2409–2427 (2023). 36858962 10.1080/15548627.2023.2186112 PMC10392742

[116] López-Otín, C., Blasco, M. A., Partridge, L., Serrano, M. & Kroemer, G. Hallmarks of aging: an expanding universe. *Cell* **186**, 243–278 (2023). 36599349 10.1016/j.cell.2022.11.001

[117] Zhang, X. et al. Local delivery of insulin/IGF-1 for bone regeneration: carriers, strategies, and effects. *Nanotheranostics* **4**, 242–255 (2020). 32923314 10.7150/ntno.46408 PMC7484631

[118] Dixit, M., Poudel, S. B. & Yakar, S. Effects of GH/IGF axis on bone and cartilage. *Mol. Cell Endocrinol.* **519**, 111052 (2021). 33068640 10.1016/j.mce.2020.111052 PMC7736189

[119] Li, D. et al. Dynamic control of mTORC1 facilitates bone healing in mice. *Bone* **190**, 117285 (2025). 39426581 10.1016/j.bone.2024.117285

[120] Hiraiwa, M. et al. mTORC1 activation in osteoclasts prevents bone loss in a mouse model of osteoporosis. *Front. Pharm.* **10**, 684 (2019). 10.3389/fphar.2019.00684 PMC6585391 31263418

[121] Liu, Q., Liu, C., Yang, Y., Yang, H. & Chen, J. Osteocyte-intrinsic mTORC1 signaling restrains trabecular bone accrual in mice. *J. Cell Biochem.* **119**, 8743–8749 (2018). 30160781 10.1002/jcb.27470

[122] Liu, J.-Y. et al. AMPK, a hub for the microenvironmental regulation of bone homeostasis and diseases. *J. Cell Physiol.* **239**, e31393 (2024). 39210747 10.1002/jcp.31393

[123] Park, S.-G. et al. The microbial metabolite imidazole propionate dysregulates bone homeostasis by inhibiting AMP-activated protein kinase (AMPK) signaling. *Commun. Biol.* **7**, 1644 (2024). 39695168 10.1038/s42003-024-07316-w PMC11655877

[124] Zhang, T. et al. Sirtuins mediate mitochondrial quality control mechanisms: a novel therapeutic target for osteoporosis. *Front. Endocrinol.* **14**, 1281213 (2023). 10.3389/fendo.2023.1281213 PMC10805026 38264287

[125] Zhao, H., Yu, F. & Wu, W. New perspectives on postmenopausal osteoporosis: mechanisms and potential therapeutic strategies of sirtuins and oxidative stress. *Antioxidants* **14**, 605 (2025). 40427485 10.3390/antiox14050605 PMC12108454

[126] Zou, Z. et al. Endothelin-1/endothelin B receptor signalling mediates Prx1+ skeletal stem cells senescence: a driver of osteoporotic bone loss. *J. Orthop. Transl.* **57**, 101048 (2026). 10.1016/j.jot.2026.101048 PMC12933462 41757294

[127] Liu, Y. et al. Chk2 deletion rescues bone loss and cellular senescence induced by Bmi1 deficiency via regulation of Cyp1a1. *J. Orthop. Transl.* **52**, 360–375 (2025). 10.1016/j.jot.2025.04.014 PMC12282458 40698069

[128] Yang, C. et al. The senolytic agent ABT263 ameliorates osteoporosis caused by active vitamin D insufficiency through selective clearance of senescent skeletal cells. *J. Orthop. Transl.* **49**, 107–118 (2024). 10.1016/j.jot.2024.08.012 PMC11490840 39430127

[129] Campisi, J. & d’Adda di Fagagna, F. Cellular senescence: when bad things happen to good cells. *Nat. Rev. Mol. Cell Biol.* **8**, 729–740 (2007). 17667954 10.1038/nrm2233

[130] Xu, Z. & Teixeira, M. T. The many types of heterogeneity in replicative senescence. *Yeast* **36**, 637–648 (2019). 31306505 10.1002/yea.3433 PMC6900063

[131] Toussaint, O. et al. Stress-induced premature senescence. essence of life, evolution, stress, and aging. *Ann. N.Y. Acad. Sci.* **908**, 85–98 (2000). 10911950 10.1111/j.1749-6632.2000.tb06638.x

[132] Wang, H. et al. Osteoclasts and osteoarthritis: novel intervention targets and therapeutic potentials during aging. *Aging Cell* **23**, e14092 (2024). 38287696 10.1111/acel.14092 PMC11019147

[133] Saeed, H. & Iqtedar, M. Bone marrow stromal cell (BMSC) and skeletal aging: role of telomerase enzyme. *Pak. J. Pharm. Sci.* **27**, 321–333 (2014). 24577922

[134] Kumar, N., Saraber, P., Ding, Z. & Kusumbe, A. P. Diversity of vascular niches in bones and joints during homeostasis, ageing, and diseases. *Front. Immunol.* **12**, 798211 (2021). 34975909 10.3389/fimmu.2021.798211 PMC8718446

[135] Peng, Y., Wu, S., Li, Y. & Crane, J. L. Type H blood vessels in bone modeling and remodeling. *Theranostics* **10**, 426–436 (2020). 31903130 10.7150/thno.34126 PMC6929606

[136] Liang, T.-Z. et al. Targeting the central and peripheral nervous system to regulate bone homeostasis: mechanisms and potential therapies. *Mil. Med. Res.* **12**, 13 (2025). 40108680 10.1186/s40779-025-00600-8 PMC11924829

[137] Liu, M. et al. Molecular mechanisms and therapeutic implications of the sympathetic nervous system in bone-related disorders: a brain-bone axis perspective. *Bone Res.* **13**, 98 (2025). 41326350 10.1038/s41413-025-00494-1 PMC12669603

[138] Zhang, H. et al. The roles of bone remodeling in normal hematopoiesis and age-related hematological malignancies. *Bone Res.* **11**, 15 (2023). 36918531 10.1038/s41413-023-00249-w PMC10014945

[139] Reya, T. et al. A role for Wnt signalling in self-renewal of haematopoietic stem cells. *Nature* **423**, 409–414 (2003). 12717450 10.1038/nature01593

[140] Mistry, J. J. et al. ROS-mediated PI3K activation drives mitochondrial transfer from stromal cells to hematopoietic stem cells in response to infection. *Proc. Natl. Acad. Sci. USA* **116**, 24610–24619 (2019). 31727843 10.1073/pnas.1913278116 PMC6900710

[141] Wang, J. et al. Bcl11a maintains hematopoietic stem cell function but accelerates inflammation-driven exhaustion during aging. *Sci. Immunol.* **10**, eadr2041 (2025). 40215324 10.1126/sciimmunol.adr2041

[142] Koh, B. I. et al. Adult skull bone marrow is an expanding and resilient haematopoietic reservoir. *Nature* **636**, 172–181 (2024). 39537918 10.1038/s41586-024-08163-9 PMC11618084

[143] Tao, H. et al. Urolithin A suppresses RANKL-induced osteoclastogenesis and postmenopausal osteoporosis by, suppresses inflammation and downstream NF-κB activated pyroptosis pathways. *Pharm. Res.* **174**, 105967 (2021). 10.1016/j.phrs.2021.105967 34740817

[144] Liu, R.-X. et al. Trim21 depletion alleviates bone loss in osteoporosis via activation of YAP1/β-catenin signaling. *Bone Res.* **11**, 56 (2023). 37884520 10.1038/s41413-023-00296-3 PMC10603047

[145] Chen, L. et al. Cross-sectional studies of the causal link between asthma and osteoporosis: insights from Mendelian randomization and bioinformatics analysis. *Osteoporos. Int.* **35**, 1007–1017 (2024). 38430243 10.1007/s00198-024-07037-0

[146] Ambrosi, T. H. et al. Aged skeletal stem cells generate an inflammatory degenerative niche. *Nature* **597**, 256–262 (2021). 34381212 10.1038/s41586-021-03795-7 PMC8721524

[147] Yin, C. et al. Senescent fibroblasts drive FAP/OLN imbalance through mTOR signaling to exacerbate inflammation and bone resorption in periodontitis. *Adv. Sci.* **12**, e2409398 (2025). 10.1002/advs.202409398 PMC11831441 39716898

[148] Blacher, E. et al. Aging disrupts circadian gene regulation and function in macrophages. *Nat. Immunol.* **23**, 229–236 (2022). 34949832 10.1038/s41590-021-01083-0 PMC9704320

[149] Widjaja, A. A. et al. Inhibition of IL-11 signalling extends mammalian healthspan and lifespan. *Nature* **632**, 157–165 (2024). 39020175 10.1038/s41586-024-07701-9 PMC11291288

[150] Critchlow, A. J., Hiam, D., Williams, R., Scott, D. & Lamon, S. The role of estrogen in female skeletal muscle aging: a systematic review. *Maturitas* **178**, 107844 (2023). 37716136 10.1016/j.maturitas.2023.107844

[151] Deltourbe, L. G. et al. Steroid hormone levels vary with sex, aging, lifestyle, and genetics. *Sci. Adv.* **11**, eadu6094 (2025). 40153492 10.1126/sciadv.adu6094 PMC11952096

[152] Hage, C. & Salvatori, R. Growth hormone and aging. *Endocrinol. Metab. Clin. North Am.* **52**, 245–257 (2023). 36948778 10.1016/j.ecl.2022.10.003

[153] Bobillier, A. et al. Association of vitamin D and parathyroid hormone status with the aging-related decline of bone microarchitecture in older men: the prospective Structure of Aging Men’s Bones (STRAMBO) study. *J. Bone Min. Res.* **37**, 1903–1914 (2022). 10.1002/jbmr.4657 35880628

[154] Reid, I. R., Bolland, M. J. & Grey, A. Effects of vitamin D supplements on bone mineral density: a systematic review and meta-analysis. *Lancet* **383**, 146–155 (2014). 24119980 10.1016/S0140-6736(13)61647-5

[155] Pondel, M. Calcitonin and calcitonin receptors: bone and beyond. *Int. J. Exp. Pathol.* **81**, 405–422 (2000). 11298188 10.1046/j.1365-2613.2000.00176.x PMC2517743

[156] Martin, C. S., Cooper, M. S. & Hardy, R. S. Endogenous glucocorticoid metabolism in bone: friend or foe. *Front Endocrinol* **12**, 733611 (2021). 10.3389/fendo.2021.733611 PMC8429897 34512556

[157] Zhu, S. et al. Endocrine regulation on bone by thyroid. *Front Endocrinol* **13**, 873820 (2022). 10.3389/fendo.2022.873820 PMC9020229 35464058

[158] Upadhyay, J., Farr, O. M. & Mantzoros, C. S. The role of leptin in regulating bone metabolism. *Metabolism* **64**, 105–113 (2015). 25497343 10.1016/j.metabol.2014.10.021 PMC4532332

[159] Naot, D., Musson, D. S. & Cornish, J. The activity of adiponectin in bone. *Calcif. Tissue Int.* **100**, 486–499 (2017). 27928591 10.1007/s00223-016-0216-5

[160] Rahmati, M., Nalesso, G., Mobasheri, A. & Mozafari, M. Aging and osteoarthritis: central role of the extracellular matrix. *Ageing Res. Rev.* **40**, 20–30 (2017). 28774716 10.1016/j.arr.2017.07.004

[161] Nyström, A. The intertwined aging, the extracellular matrix, and fibrosis. *Am. J. Physiol. Cell Physiol.* **326**, C645–C646 (2024). 38223933 10.1152/ajpcell.00018.2024

[162] Zhang, L., Zhou, J. & Kong, W. Extracellular matrix in vascular homeostasis and disease. *Nat. Rev. Cardiol.* **22**, 333–353 (2025). 39743560 10.1038/s41569-024-01103-0

[163] Yi, J. et al. Elastin-derived extracellular matrix fragments drive aging through innate immune activation. *Nat. Aging* **5**, 2380–2398 (2025). 41023316 10.1038/s43587-025-00961-8 PMC12705442

[164] Boaru, D. L. et al. Extracellular matrix dysregulation in aging, calcification, and cancer diseases: insights into cellular senescence, inflammation, and novel therapeutic strategies. *Int. J. Biol. Sci.* **21**, 6808–6881 (2025). 41281748 10.7150/ijbs.119301 PMC12631191

[165] Garg, K. & Boppart, M. D. Influence of exercise and aging on extracellular matrix composition in the skeletal muscle stem cell niche. *J. Appl. Physiol.* **121**, 1053–1058 (2016). 27539500 10.1152/japplphysiol.00594.2016 PMC5142247

[166] Guvatova, Z. G., Borisov, P. V., Alekseev, A. A. & Moskalev, A. A. Age-related changes in extracellular matrix. *Biochemistry* **87**, 1535–1551 (2022). 36717445 10.1134/S0006297922120112

[167] Bab, I., Smoum, R., Bradshaw, H. & Mechoulam, R. Skeletal lipidomics: regulation of bone metabolism by fatty acid amide family. *Br. J. Pharm.* **163**, 1441–1446 (2011). 10.1111/j.1476-5381.2011.01474.x PMC3165954 21557736

[168] Khazai, N., Judd, S. E. & Tangpricha, V. Calcium and vitamin D: skeletal and extraskeletal health. *Curr. Rheumatol. Rep.* **10**, 110–117 (2008). 18460265 10.1007/s11926-008-0020-y PMC2669834

[169] Massé, O. et al. Calcium, vitamin D, or combined supplementation to prevent fractures and falls: systematic review and meta-analysis. *BMJ* **393**, e088050 (2026). 42161415 10.1136/bmj-2025-088050 PMC13188451

[170] Aaseth, J. O., Finnes, T. E., Askim, M. & Alexander, J. The importance of vitamin K and the combination of vitamins K and D for calcium metabolism and bone health: a review. *Nutrients* **16**, 2420 (2024). 39125301 10.3390/nu16152420 PMC11313760

[171] Rizzoli, R. & Chevalley, T. Nutrition and osteoporosis prevention. *Curr. Osteoporos. Rep.* **22**, 515–522 (2024). 39322861 10.1007/s11914-024-00892-0 PMC11499541

[172] Zhang, J. et al. Feeding blueberry diets in early life prevent senescence of osteoblasts and bone loss in ovariectomized adult female rats. *PLoS One* **6**, e24486 (2011). 21912699 10.1371/journal.pone.0024486 PMC3166322

[173] Li, T., Wu, S.-M., Xu, Z.-Y. & Ou-Yang, S. Rabbiteye blueberry prevents osteoporosis in ovariectomized rats. *J. Orthop. Surg. Res* **9**, 56 (2014). 25102951 10.1186/s13018-014-0056-9 PMC4237865

[174] Damani, J. J., De Souza, M. J., VanEvery, H. L., Strock, N. C. A. & Rogers, C. J. The role of prunes in modulating inflammatory pathways to improve bone health in postmenopausal women. *Adv. Nutr.* **13**, 1476–1492 (2022). 34978320 10.1093/advances/nmab162 PMC9526830

[175] Damani, J. J. et al. Prune Consumption attenuates proinflammatory cytokine secretion and alters monocyte activation in postmenopausal women: secondary outcome analysis of a 12-mo randomized controlled trial: the Prune study. *J. Nutr.* **154**, 1699–1710 (2024). 37984741 10.1016/j.tjnut.2023.11.014 PMC11347809

[176] Hooshmand, S. et al. The effect of two doses of dried plum on bone density and bone biomarkers in osteopenic postmenopausal women: a randomized, controlled trial. *Osteoporos. Int.* **27**, 2271–2279 (2016). 26902092 10.1007/s00198-016-3524-8

[177] Smith, B. J. et al. Montmorency tart cherry protects against age-related bone loss in female C57BL/6 mice and demonstrates some anabolic effects. *Eur. J. Nutr.* **58**, 3035–3046 (2019). 30377814 10.1007/s00394-018-1848-1

[178] Dodier, T. et al. U.S. Montmorency tart cherry juice decreases bone resorption in women aged 65-80 Years. *Nutrients* **13**, 544 (2021). 33562341 10.3390/nu13020544 PMC7914470

[179] Adedigba, P. et al. Dietary tart cherry and fructooligosaccharides promote bone health via the gut microbiota and increased bone formation. *Nutrients* **17**, 2829 (2025). 40944216 10.3390/nu17172829 PMC12430659

[180] Shimizu, S. et al. Effect of anthocyanin-rich bilberry extract on bone metabolism in ovariectomized rats. *Biomed. Rep.* **8**, 198–204 (2018). 29435281 10.3892/br.2017.1029 PMC5776409

[181] Perna, S. et al. Berries derived polyphenols and bone health: a systematic review. *Nutrients* **17**, 3440 (2025). 41228518 10.3390/nu17213440 PMC12610276

[182] New, S. A. et al. Dietary influences on bone mass and bone metabolism: further evidence of a positive link between fruit and vegetable consumption and bone health?. *Am. J. Clin. Nutr.* **71**, 142–151 (2000). 10617959 10.1093/ajcn/71.1.142

[183] Langsetmo, L. et al. Dietary patterns and incident low-trauma fractures in postmenopausal women and men aged ≥50 y: a population-based cohort study. *Am. J. Clin. Nutr.* **93**, 192–199 (2011). 21068350 10.3945/ajcn.110.002956 PMC5101071

[184] Rizzoli, R. & Chevalley, T. Bone health: biology and nutrition. *Curr. Opin. Clin. Nutr. Metab. Care* **27**, 24–30 (2024). 37922025 10.1097/MCO.0000000000000988 PMC10720787

[185] LeBoff, M. S. & Bischoff-Ferrari, H. A. The effects of vitamin D supplementation on musculoskeletal health: The VITAL and DO-health trials. *J. Gerontol. A Biol. Sci. Med. Sci.* **78**, 73–78 (2023). 37325962 10.1093/gerona/glad073 PMC10272981

[186] Mullath Ullas, A., Boamah, J., Hussain, A., Myrtziou, I. & Kanakis, I. Impact of dietary patterns on skeletal health: a systematic review and meta-analysis of bone mineral density, fracture, bone turnover markers, and nutritional status. *Nutrients* **17**, 3845 (2025). 41470790 10.3390/nu17243845 PMC12735435

[187] Willems, H. M. E., van den Heuvel, E. G. H. M., Schoemaker, R. J. W., Klein-Nulend, J. & Bakker, A. D. Diet and exercise: a match made in bone. *Curr. Osteoporos. Rep.* **15**, 555–563 (2017). 29098573 10.1007/s11914-017-0406-8 PMC5705732

[188] Shojaa, M. et al. Effects of dynamic resistance exercise on bone mineral density in postmenopausal women: a systematic review and meta-analysis with special emphasis on exercise parameters. *Osteoporos. Int.* **31**, 1427–1444 (2020). 32399891 10.1007/s00198-020-05441-w PMC7360540

[189] Mello, J. B. et al. Exercise in school physical education increase bone mineral content and density: systematic review and meta-analysis. *Eur. J. Sport Sci.* **22**, 1618–1629 (2022). 34328066 10.1080/17461391.2021.1960426

[190] Wilson, D. J. Osteoporosis and sport. *Eur. J. Radiol.* **110**, 169–174 (2019). 30599856 10.1016/j.ejrad.2018.11.010

[191] Di Lorito, C. et al. Exercise interventions for older adults: a systematic review of meta-analyses. *J. Sport Health Sci.* **10**, 29–47 (2021). 32525097 10.1016/j.jshs.2020.06.003 PMC7858023

[192] Kemmler, W., Shojaa, M., Kohl, M. & von Stengel, S. Effects of different types of exercise on bone mineral density in postmenopausal women: a systematic review and meta-analysis. *Calcif. Tissue Int.* **107**, 409–439 (2020). 32785775 10.1007/s00223-020-00744-w PMC7546993

[193] Zhu, T.-R. et al. Effectiveness of exercise prescription variables to reduce fall risk among older adults: a meta-analysis. *Eur. Rev. Aging Phys. Act.* **22**, 7 (2025). 40360991 10.1186/s11556-025-00374-x PMC12070723

[194] Zitzmann, A.-L. et al. The effect of different training frequency on bone mineral density in older adults. A comparative systematic review and meta-analysis. *Bone* **154**, 116230 (2022). 34624560 10.1016/j.bone.2021.116230

[195] Balaji, S. M. Effect of smoking on implant-bone interface. *Indian J. Dent. Res.* **30**, 3 (2019). 30900647 10.4103/ijdr.IJDR_140_19

[196] Truntzer, J., Vopat, B., Feldstein, M. & Matityahu, A. Smoking cessation and bone healing: optimal cessation timing. *Eur. J. Orthop. Surg. Traumatol.* **25**, 211–215 (2015). 24879610 10.1007/s00590-014-1488-y

[197] Shin, M. et al. Impact of heavy alcohol consumption on cortical bone mechanical properties in male rhesus macaques. *Bone* **181**, 117041 (2024). 38325648 10.1016/j.bone.2024.117041 PMC12364445

[198] Sampson, H. W. Alcohol’s harmful effects on bone. *Alcohol Health Res. World* **22**, 190–194 (1998). 15706795 PMC6761900

[199] Cheraghi, Z. et al. The effect of alcohol on osteoporosis: a systematic review and meta-analysis. *Drug Alcohol Depend.* **197**, 197–202 (2019). 30844616 10.1016/j.drugalcdep.2019.01.025

[200] Khiyali, Z., Rashedi, V., Tavacol, Z., Dehghan, A. & Bijani, M. Smoking, alcohol consumption, drug abuse, and osteoporosis among older adults: a cross-sectional study on PERSIAN cohort study in Fasa. *BMC Geriatr.* **24**, 80 (2024). 38254032 10.1186/s12877-024-04678-y PMC10802063

[201] Aibar-Almazán, A. et al. Current status of the diagnosis and management of osteoporosis. *Int. J. Mol. Sci.* **23**, 9465 (2022). 36012730 10.3390/ijms23169465 PMC9408932

[202] Management of osteoporosis in postmenopausal women: the 2021 position statement of The North American Menopause Society. *Menopause* **28**, 973–997 (2021). 10.1097/GME.0000000000001831 34448749

[203] Ayers, C. et al. Effectiveness and safety of treatments to prevent fractures in people with low bone mass or primary osteoporosis: a living systematic review and network meta-analysis for the American College of Physicians. *Ann. Intern. Med.* **176**, 182–195 (2023). 36592455 10.7326/M22-0684

[204] Curtis, J. R. et al. Comparative effectiveness of denosumab vs alendronate among postmenopausal women with osteoporosis. *J. Bone Miner. Res.* **39**, 826–834 (2024). 38753892 10.1093/jbmr/zjae079 PMC11301726

[205] Nakafero, G. et al. Fragility fracture, atypical femoral fracture, and osteonecrosis of jaw after bisphosphonate prescription for three and five years, based on primary and secondary care data in England: nested case-control and cohort studies. *BMJ Med.* **5**, e002085 (2026). 42046648 10.1136/bmjmed-2025-002085 PMC13110635

[206] Kendler, D. L., Cosman, F., Stad, R. K. & Ferrari, S. Denosumab in the treatment of osteoporosis: 10 years later: a narrative review. *Adv. Ther.* **39**, 58–74 (2022). 34762286 10.1007/s12325-021-01936-y PMC8799550

[207] Kumar, S. et al. Denosumab discontinuation in the clinic: implications of rebound bone turnover and emerging strategies to prevent bone loss and fractures. *J. Bone Miner. Res.* **40**, 1017–1034 (2025). 40057981 10.1093/jbmr/zjaf037 PMC12406127

[208] Degboé, Y. & Couture, G. Strategies for denosumab discontinuation in postmenopausal osteoporosis. *Jt. Bone Spine* **93**, 105954 (2026). 10.1016/j.jbspin.2025.105954 40885300

[209] Menéndez, J. Q., García Tellado, Á, Pardo Lleidas, J. & Hernández Hernández, J. L. Hypercalcemia after denosumab discontinuation in patients with osteoporosis: a systematic review and case report. *Osteoporos. Int.* **37**, 637–642 (2026). 41571922 10.1007/s00198-026-07854-5 PMC13083444

[210] Shogry, F. F. et al. The rise and fall of raloxifene use for osteoporosis, 1999-2022. *Osteoporos. Int.* **36**, 1277–1286 (2025). 40498127 10.1007/s00198-025-07544-8 PMC12403046

[211] Muñoz-Torres, M., Alonso, G. & Raya, M. P. Calcitonin therapy in osteoporosis. *Treat. Endocrinol.* **3**, 117–132 (2004). 15743107 10.2165/00024677-200403020-00006

[212] Luo, Y., Liang, M., Gao, Y., Wang, X. & Li, Y. Clinical indicators for perimenopausal hormone replacement therapy: an evidence-based narrative review. *Niger. J. Clin. Pract.* **29**, 373–381 (2026). 42085078 10.4103/njcp.njcp_879_25

[213] Lorentzon, M. et al. Menopausal hormone therapy reduces the risk of fracture regardless of falls risk or baseline FRAX probability—results from the Women’s Health Initiative hormone therapy trials. *Osteoporos. Int.* **33**, 2297–2305 (2022). 35833956 10.1007/s00198-022-06483-y PMC9568435

[214] Teriparatide. in *Drugs and Lactation Database (LactMed®)* (National Institute of Child Health and Human Development, 2006).

[215] Vahle, J. L. et al. Skeletal changes in rats given daily subcutaneous injections of recombinant human parathyroid hormone (1-34) for 2 years and relevance to human safety. *Toxicol. Pathol.* **30**, 312–321 (2002). 12051548 10.1080/01926230252929882

[216] Gilsenan, A. et al. Teriparatide did not increase adult osteosarcoma incidence in a 15-year US postmarketing surveillance study. *J. Bone Miner. Res.* **36**, 244–251 (2021). 32990990 10.1002/jbmr.4188 PMC7988650

[217] Barnett, M., Riley, L. J., Casipit, C. & Azmaiparashvili, Z. Boxed in: should the black box warning for osteosarcoma be retired for good? *Endocr. Abstr.* **110**, (2025).

[218] Miller, P. D. et al. Effect of abaloparatide vs placebo on new vertebral fractures in postmenopausal women with osteoporosis: a randomized clinical trial. *JAMA* **316**, 722–733 (2016). 27533157 10.1001/jama.2016.11136

[219] Wu, D., Li, L., Wen, Z. & Wang, G. Romosozumab in osteoporosis: yesterday, today and tomorrow. *J. Transl. Med.* **21**, 668 (2023). 37759285 10.1186/s12967-023-04563-z PMC10523692

[220] Maruyama, H. et al. Cardiovascular risk of romosozumab vs. teriparatide: a cohort study using Japan’s National Claims Database. *Clin. Pharm. Ther.* **119**, 721–728 (2026). 10.1002/cpt.70142 PMC12882750 41272927

[221] Cheng, S.-H., Chu, W., Chou, W.-H., Chu, W.-C. & Kang, Y.-N. Cardiovascular safety of romosozumab compared to commonly used anti-osteoporosis medications in postmenopausal osteoporosis: a systematic review and network meta-analysis of randomized controlled trials. *Drug Saf.* **48**, 7–23 (2025). 39227560 10.1007/s40264-024-01475-9 PMC11711713

[222] Nayak, S. & Greenspan, S. L. A systematic review and meta-analysis of sequential treatment strategies for osteoporosis. *Osteoporos. Int.* **37**, 1–13 (2026). 41105226 10.1007/s00198-025-07717-5

[223] Cipolloni, V. et al. The anabolic-first strategy in osteoporosis: a systematic review and meta-analysis of fracture outcomes in patients at very high fracture risk. *Medicines* **62**, 687 (2026). 10.3390/medicina62040687 PMC13117170 42075559

[224] Reid, I. R. & Billington, E. O. Drug therapy for osteoporosis in older adults. *Lancet* **399**, 1080–1092 (2022). 35279261 10.1016/S0140-6736(21)02646-5

[225] Kirkland, J. L. & Tchkonia, T. Senolytic drugs: from discovery to translation. *J. Intern. Med.* **288**, 518–536 (2020). 32686219 10.1111/joim.13141 PMC7405395

[226] Ansari, M. M., Ghosh, M., Lee, D.-S. & Son, Y.-O. Senolytic therapeutics: an emerging treatment modality for osteoarthritis. *Ageing Res. Rev.* **96**, 102275 (2024). 38494091 10.1016/j.arr.2024.102275

[227] Zhu, Y. et al. The Achilles’ heel of senescent cells: from transcriptome to senolytic drugs. *Aging Cell* **14**, 644–658 (2015). 25754370 10.1111/acel.12344 PMC4531078

[228] Farr, J. N. et al. Effects of intermittent senolytic therapy on bone metabolism in postmenopausal women: a phase 2 randomized controlled trial. *Nat. Med.* **30**, 2605–2612 (2024). 38956196 10.1038/s41591-024-03096-2 PMC11705617

[229] Houssaini, A. et al. mTOR pathway activation drives lung cell senescence and emphysema. *JCI Insight* **3**, e93203 (2018). 93203. 29415880 10.1172/jci.insight.93203 PMC5821218

[230] Yousefzadeh, M. J. et al. Fisetin is a senotherapeutic that extends health and lifespan. *EBioMedicine* **36**, 18–28 (2018). 30279143 10.1016/j.ebiom.2018.09.015 PMC6197652

[231] Lin, C. et al. Engineering a targeted and safe bone anabolic gene therapy to treat osteoporosis in alveolar bone loss. *Mol. Ther.* **32**, 3080–3100 (2024). 38937970 10.1016/j.ymthe.2024.06.036 PMC11403231

[232] Ocarino de, N. M. et al. Intra-bone marrow injection of mesenchymal stem cells improves the femur bone mass of osteoporotic female rats. *Connect Tissue Res.* **51**, 426–433 (2010). 20373890 10.3109/03008201003597049

[233] Cao, C. et al. Bone marrow mesenchymal stem cells slow intervertebral disc degeneration through the NF-κB pathway. *Spine J.* **15**, 530–538 (2015). 25457469 10.1016/j.spinee.2014.11.021

[234] Kostecka, A. et al. Adipose-derived mesenchymal stromal cells in clinical trials: insights from single-cell studies. *Life Sci.* **351**, 122761 (2024). 38866216 10.1016/j.lfs.2024.122761

[235] Evans, C. H., Ghivizzani, S. C. & Robbins, P. D. Orthopaedic gene therapy: twenty-five years on. *JBJS Rev.* **9** (2021). 10.2106/JBJS.RVW.20.00220 PMC8654240 34437305

[236] Oh, W.-T. et al. WNT-modulating gene silencers as a gene therapy for osteoporosis, bone fracture, and critical-sized bone defects. *Mol. Ther.* **31**, 435–453 (2023). 36184851 10.1016/j.ymthe.2022.09.018 PMC9931550

[237] Serrano, D. R. et al. Exosome-based drug delivery: a next-generation platform for cancer, infection, neurological and immunological diseases, gene therapy and regenerative medicine. *Pharmaceutics* **17**, 1336 (2025). 41155971 10.3390/pharmaceutics17101336 PMC12567338

[238] Jakob, F. et al. Bone tissue engineering in osteoporosis. *Maturitas* **75**, 118–124 (2013). 23562167 10.1016/j.maturitas.2013.03.004

[239] de Moraes, V. A. F., Moreira, F. M., Dos Santos Santinoni, C., de Souza Batista, V. E. & Mori, G. G. Would exosome therapy be effective for bone regeneration? Systematic review and meta-analysis. *Calcif. Tissue Int.* **116**, 127 (2025). 41077581 10.1007/s00223-025-01438-x

[240] Zhang, Q. et al. Tissue engineering and regenerative medicine therapies for cell senescence in bone and cartilage. *Tissue Eng. Part. B. Rev.* **26**, 64–78 (2020). 31801419 10.1089/ten.TEB.2019.0215

